# Neurorehabilitation Clinical Pathways in Stroke: The Padova Model

**DOI:** 10.1007/s10072-025-08770-y

**Published:** 2026-02-13

**Authors:** Alessandra Del Felice, Antonio Luigi Bisogno, Silvia Facchini, Serena De Pellegrin, Alfonc Baba, Alex Lando, Anna Maria Basile, Francesco Piccione, Angelo Antonini, Corbetta Maurizio

**Affiliations:** 1 Dipartimento di Neuroscienze, University of Padova, Padua, Italy; 2 Padova Neuroscience Center, University of Padova, Padua, Italy; 3 Azienda Ospedale Università di Padova, Padua, Italy; 4 Department of Biomedical and Neuromotor Sciences, Alma Mater University of Bologna, Bologna, Italy; 5 IRCCS San Camillo Hospital, Venice, Italy; 6 Veneto Institute of Molecular Medicine (VIMM), Padova, Italy

**Keywords:** Rehabilitation, Rehabilitative pathways, Health organization, Evidence-based medicine

## Abstract

**Background:**

The rising prevalence of neurological disabilities underscores the need for neurorehabilitation units grounded in evidence-based medicine. However, a gap persists between available evidence and its consistent implementation in clinical practice. In this study, we present the experience of a single tertiary medical center and propose a structured, evidence-based framework for stroke neurorehabilitation aimed at standardizing care and improving functional outcomes.

**Methods:**

An iterative consensus process adapted from the modified Delphi Panel methodology was conducted. A multidisciplinary panel of clinicians and experts in stroke and neurorehabilitation reviewed evidence-based guidelines addressing upper limb rehabilitation, gait and balance, cognitive rehabilitation, speech and language therapy, and dysphagia. Guidelines were identified through systematic searches of databases, professional society websites, and reference lists, and appraised using the AGREE II instrument by three independent evaluators. Consensus was achieved through remote feedback rounds and structured in-person meetings between November 2023 and March 2024. Agreement was defined as ≥75% panel consensus.

**Results:**

Fifty-two guidelines were identified; eight were excluded due to low methodological quality (AGREE II score <6/7). Consensus was reached across all rehabilitation domains through one to three iterative rounds, depending on topic complexity. Agreement was achieved on all proposed assessment tools and intervention items for upper limb, gait and balance, cognitive, speech and language, and dysphagia rehabilitation. Based on consensus outputs, interventions were stratified according to two hierarchical dimensions: behavioral deficits and impairment severity (mild–moderate vs severe), informed by clinical and instrumental assessments and aligned with the International Classification of Functioning, Disability, and Health framework.

**Conclusions:**

Using a structured, multidisciplinary consensus approach, we developed evidence-based and context-sensitive neurorehabilitation clinical pathways supported by pragmatic stratification algorithms. This framework provides a reproducible model for translating guideline evidence into standardized inpatient neurorehabilitation practice, with the potential to optimize functional outcomes and quality of life in individuals with neurological disabilities.

**Supplementary Information:**

The online version contains supplementary material available at 10.1007/s10072-025-08770-y.

## Introduction

Disability related to neurological disorders is steadily increasing. In 2021, it was the leading cause of disability adjusted life years (DALYs, 443 million), affecting 3.40 billion individuals, or 43.1% of the global population. Among this group, stroke was the condition with the highest age-adjusted DALYs. This burden calls for adequate care pathways, starting from prevention and acute care to neurorehabilitation [1].

Neurorehabilitation entails the process of recovery of function and reintegration in the social/working context. In Western countries, it is a constituting component of care and affected individuals and caregivers place great expectations in the process. The need for evidence-based, cost-effective neurorehabilitation pathways is thus utmost but their development and uptake present challenges.

Scientific evidence in neurorehabilitation, once scarce, has increased in recent years. In fact, systematic reviews offer an in-depth analysis of existing evidence but are mainly focused on a single topic and do not provide practical indication on the clinical transferability of results [2]. Guidelines and consensus-based recommendations [3] offer a wider perspective but often focus on a single disability (i.e. motor, speech or language), lacking an integrated perspective on multiple simultaneously occurring impairments, which is the rule rather than the exception in neurological disorders including stroke [4–8].

The adoption of evidence-based methods is frequently hindered by a lack of adequate training or expertise in specific techniques, the complexity of intervention protocols, and beliefs about effectiveness, as well as lack of accountability perceived by therapists [9].

Clinical pathways (CPs) are an attempt to overcome these limitations. CPs are organized, multi-professional care plans for specific medical conditions [10]. They outline a standardized approach for managing complex care processes at a particular healthcare facility, with consistent, evidence-based care across healthcare teams and settings. CPs are characterized by a structured, multidisciplinary care plan, adapting evidence or guidelines to local settings, and standardizing it. They provide a detailed outline of treatment steps for a specific medical condition and a timeline with assessment-based milestones for progression [11].

At the University Hospital of Padova, we implemented a practical clinical pathway for rehabilitation of disability related to neurological disorders, based on up-to-date evidence. A strong multidisciplinary and translational effort ensured a systemic approach, considering impairment in the frame of diverse neurological disorders and incorporating the complex interaction of motor and cognitive deficits. The use of technologies for assessment and training supported stratification based on timing and severity of impairment to personalize treatment.

The outputs are practical flow charts of the best-evidence assessment and treatment options. These flowcharts will be implemented in the future in the local neurorehabilitation unit. An innovation inspired working culture will ensure their implementation and uptake.

## Methods

We used an iterative approach adapted from the modified Delphi Panel methodology [12, 13]. The problem area was identified by a first open ended discussion round focusing on evidence-based neurorehabilitation interventions in clinical practice. This first meeting highlighted the need for evidence-based protocols, including assessment and treatment, to be adopted by the whole neurorehabilitation team at the University Hospital of Padova. Based on the Unit organization, which includes a Stroke Unit and an acute and a subacute Neurology ward, the group excluded from the discussion disorders of consciousness and out-patient/home-based rehabilitation.

The panel consisted of clinicians and experts in the field of neurorehabilitation at the University and University Hospital of Padova. This multidisciplinary group included experts in stroke neurorehabilitation, speech and language therapy, cognitive rehabilitation, and physiotherapy. We did not identify any conflict of interest among the panelists. The same panel achieved consensus by following a three-stage process. The decision-making process consisted of a first round of remote feedback on a review of each topic followed by one in person meeting for each topic (from November 2023 to March 2024). A summary of outputs and the review of the first draft of the article were discussed in a final in-person meeting.

Before each meeting of the Padova Neurorehabilitation group, the experts performed a review of the available guidelines. The topics were: upper limb rehabilitation, gait & balance rehabilitation, cognitive rehabilitation, speech & language rehabilitation, dysphagia rehabilitation. Query strings are reported in the Supplementary material (see S1. Query strings). In addition, bibliographies and websites of the main scientific associations of stroke/cerebrovascular disease/Parkinson’s disease/physiotherapy/rehabilitation societies were scanned to include missing guidelines. After identification of the papers, these were appraised following the AGREE II methodology by three independent evaluators (ALB, AB, DBV) [14]. Adaptation to the local context was performed during face-to-face meetings. To reduce dominance and group conformity, the summary of each literature revision, presented as slides, was shared among all group participants before meetings and structured feedback requested. Feedback was a binary choice—agree, disagree on question X on a specific topic. Frequencies of disagreement for each topic were obtained. Reasons for disagreement were discussed in face-to-face meetings, organized with a bimonthly schedule.

**Fig. 1:**
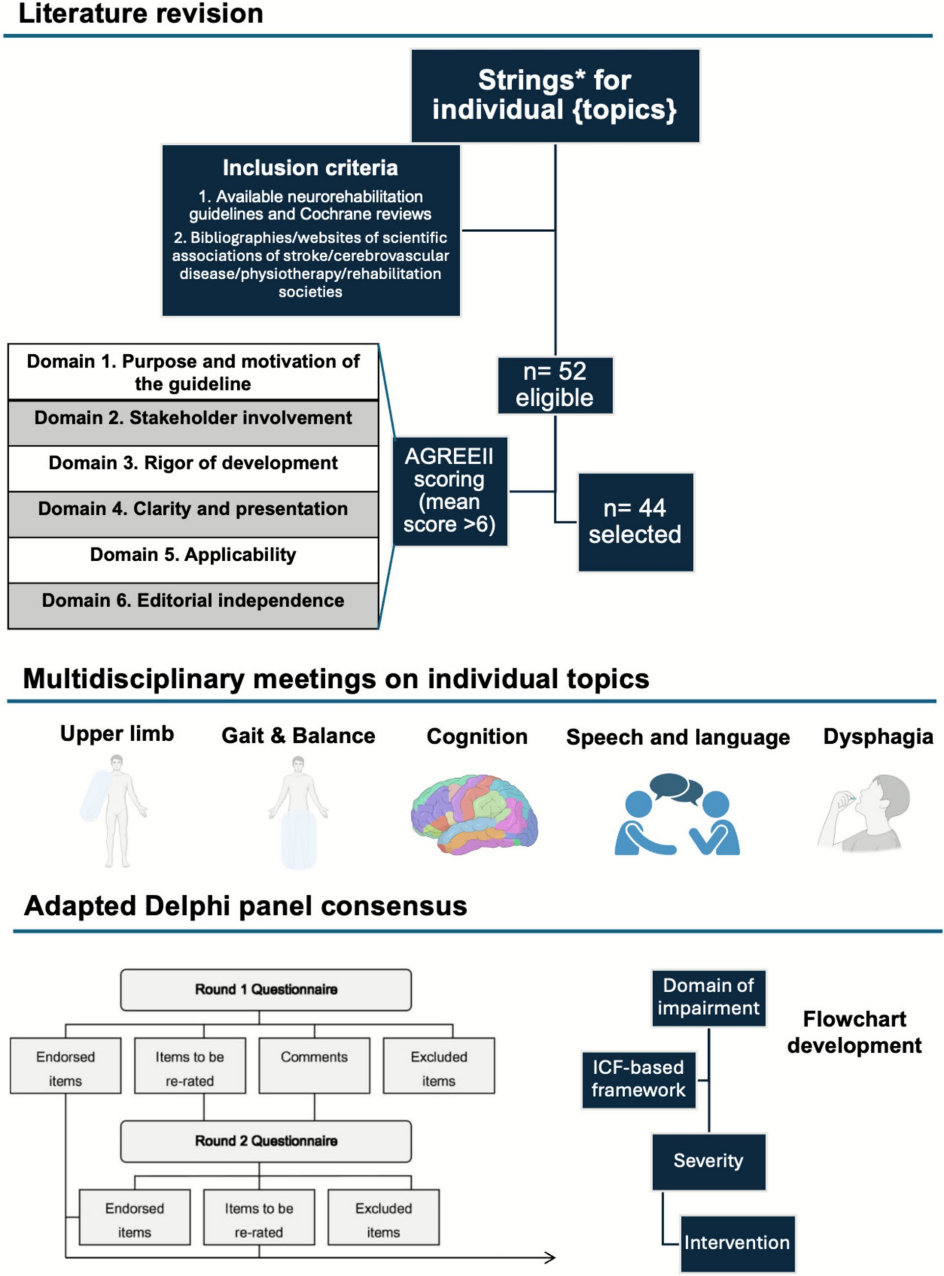
Methodological framework. A literature revision identified relevant neurorehabilitation guidelines for upper limb, gait&balance, cognitive, speech & and language and dysphagia rehabilitation. Multidisciplinary meetings on the individual topics followed. An adapted Delphi panel identified relevant items that were included in practical flowcharts of interventions

Agreement was reached by consensus, defined as 75% of panel members [15]. In case of disagreement, the indications of all available guidelines on the specific topic were re-reviewed and inputs expressed by panelist in the previous incorporated in the updated version of the recommendation; the assessment/intervention reported in the highest number of guidelines were re-discussed considering local adaptations. Figure 1 describes our methodological framework.

## Results

We identified 50 guidelines (a complete list can be found in S2. Neurorehabilitation guidelines). The mean AGREEII scores for all guidelines was 6.67 ± 0.42. We excluded 10 guidelines scoring less than 6 out of 7.

Upper limb (UL) rehabilitation consensus was reached at first round for 30 items out of 30 by a panel of 7 experts (MC, ADF, FP, AM, AA, AB, DV). For gait and balance rehabilitation, agreement was reached at first round for 27 items out of 27 by the same panel of UL rehabilitation. Five panelists (SDP, IB, MC, AMB, VV) reviewed speech and language rehabilitation items. 15 items out of 17 reached agreement after first round, with the remaining two reaching consensus after the second round. Seven panelists (MC, PB, MB, SF, MD, FM, AMB) evaluated cognitive rehabilitation items. Agreement was reached at first round for 16 items out of 20. Agreement was reached at second round for 2 items out of 4. Agreement was reached at third round for 2 items out of 2. Finally, seven panelists responded to dysphagia (SDP, SM, EB, CF, AF, IB, GM) rehabilitation items. Agreement was reached at first round for 2 items, with the final round providing agreement on the last 3 items.

## Stratification method

**Fig. 2:**
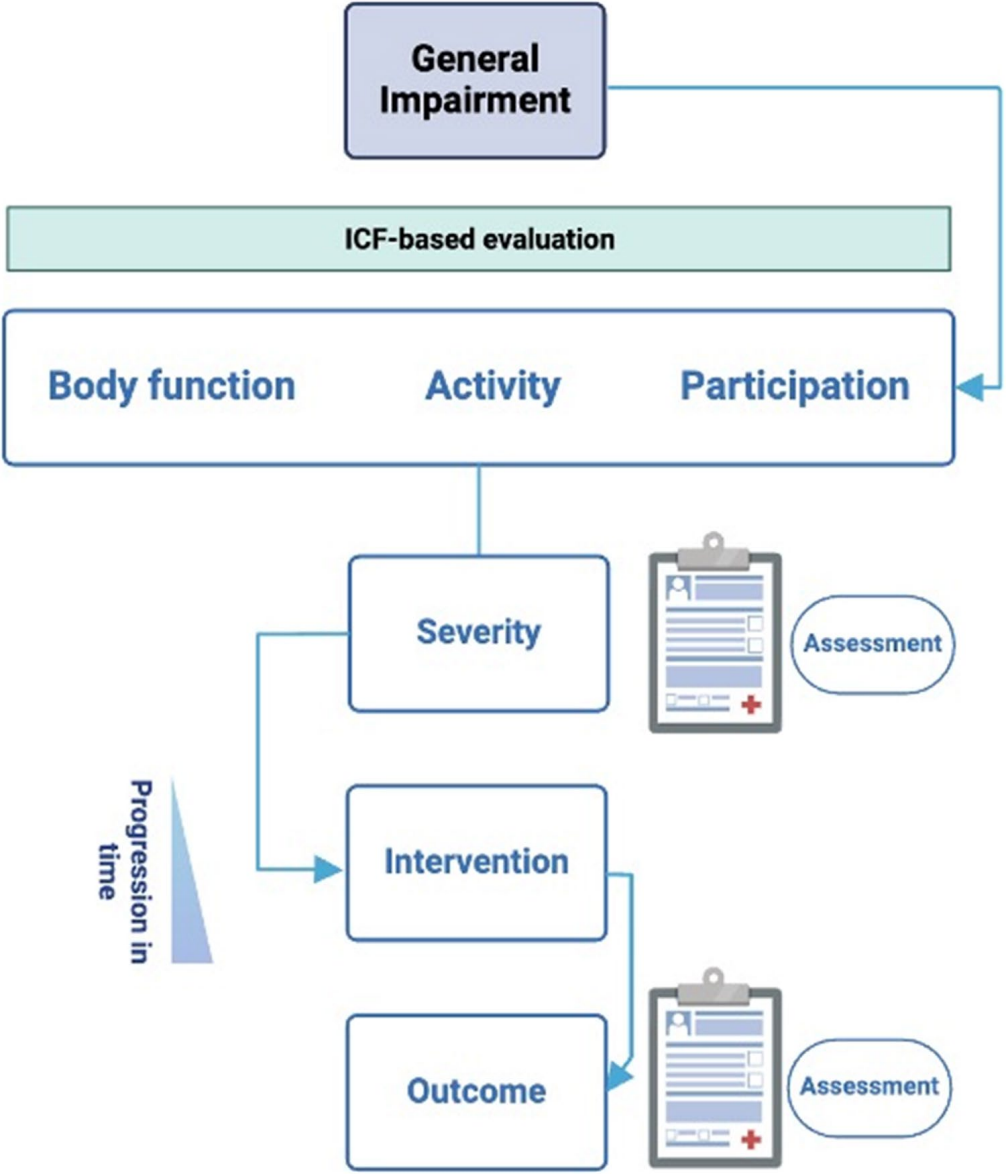
Stratification approach for personalized intervention and assessment. Impairment is classified based on the ICF framework. Clinical and instrumental assessment provide stratification based on severity and provide milestones for temporal progression of interventions

We stratified interventions based on two hierarchical levels: behavioral deficits and impairment severity. This structured, pragmatic approach ensures rehabilitation strategies are tailored to meet the individual needs based on sound clinical/instrumental assessments. The expert group chose this behavioral pragmatic approach to ensure relevance of interventions for effective functional gain. Stratifying by severity and selecting interventions based on a temporal progression, which mirrors gradual recovery or worsening of function, ensures that rehabilitation efforts align with the person’s current abilities (see Fig. 2 for a graphical representation of this stratification method). This approach builds on the International Classification of Functioning, Disability, and Health (ICF) framework perspective [16]. According to ICF, three domains define health and well-being: Body Function, Activity, and Participation. Impairments are detailed for each specific domain of function (i.e., strength and coordination for upper limb, or gait and balance for lower limb). Severity is stratified into mild to moderate or severe based on common clinical assessments and instrumental assessments whenever available. While we chose to focus on clinical evaluations commonly used in real-world settings, instrumental assessments are recommended to complement this classification if available (i.e. neurophysiology, kinematics or advanced neuroimaging metrics). Lastly, the diagram also incorporates a progression timeline, which is visually represented as a gradient at the bottom left of the chart. We included an additional etiological level only in the case of speech and language rehabilitation. This decision was driven by the fact that we identified only for this domain of function a clear distinction of interventions (i.e. the temporal progression of interventions) based on aetiology, according to available guidelines [17, 18].

## Evidence-based assessment and intervention

### Upper limb rehabilitation

#### Clinical assessment

We provide a concise summary of clinical assessments for upper limb impairment severity and outcome evaluation. This is a pragmatic selection based on the screened guidelines and clinical context (Supplementary material Table S3_Clinical assessments). These assessments are commonly used tools for evaluating various aspects of physical function and disability, classified under the ICF framework.

Under the body function domain, we selected the following scales: the Action Research Arm Test (ARAT) [19], the Fugl-Meyer Assessment for the Upper Limb (Fugl-Meyer UL, including the sensory function subsection, specifically proprioception) [20], the Medical Research Council (MRC), [21] and the Modified Ashworth Scale (MAS) [22] scale are used to evaluate strength, coordination, and spasticity. The Unified Parkinson's Disease Rating Scale (UPDRS) [23] assesses motility, bradykinesia and tremor, and falls both under body function and activity domain.

It shall be emphasized that lesions affecting the central motor pathways (primary motor, association cortex, cortico-spinal tract) affect primarily movements that are behaviorally relevant and organized in multi-segment actions (motor synergies), while lesions affecting spinal cord, nerves, muscles can present as more focal motor deficits. Hence in terms of assessment it is important to use functional tasks like the ARAT for central lesions and the MRC for peripheral lesions. It should be also emphasized that movement involves cognition in terms of attention and planning, hence assessments for apraxia [24] and motor neglect [25] shall be also part of the evaluation of a patient who does not move well the upper extremity. ARAT and Fugl-Meyer measure function and impairment according to the ICF classification, but their scores are highly correlated. The Fugl-Meyer is widely used in research work on motor recovery.

The modified Rankin Scale (mRS) assesses overall disability and is classified under activity and participation, reflecting a person’s ability to carry out daily tasks and engage in social roles. Despite its coarse range (0 = no symptoms, 1 = symptoms, but no disability; 2 = slight disability but able to look after own affairs with no assistance; 3 = moderate disability but able to walk without assistance; 4 = moderate severe disability, unable to walk without assistance but independent for bodily functions; 5 = severe disability, bedridden, incontinence; 6 = death) it is the scale more widely used in acute stroke treatment trials. Typically, a significant change after treatment in the percentage of people with scores 1–2 is considered effective reflecting a strong behavioral effect. However, most disability in stroke is cognitive. The Stroke Impact Scale (SIS) is a comprehensive tool that covers body function, activity, communication, and social participation, capturing the wide-ranging effects of stroke on an individual's life. Importantly, the SIS can be carried out through a phone interview which facilitates long-term follow up of patients.

While the focus is on clinical evaluations of UL motor deficits, it must be noted that a thorough evaluation of severity should be complemented by instrumental assessments. Motor Evoked Potentials (MEPs) are part of the clinical and prognostic work-up of motor impairments following stroke, as they provide an indication on the residual function of the corticospinal tract and allow a proper stratification of the recovery potential. Specifically, positive MEPs at around three days post-stroke predict proportional motor recovery [26], and along with acute FMA explain about 60% of the variability of chronic motor scores [27]. The MEPs are particularly helpful in stratifying patients with poor acute motor function and lesions that on imaging affect the corticospinal tract [28, 29]. The presence of an MEP can be important to allocate proper physiotherapy resources in the acute setting [30].

The technique requires basic neurophysiological skills, but the main limiting factor is the availability of equipment. Similarly, we included kinematic assessments with commercial Inertial Motion Units (IMUs) of UL during standardized tasks (ARAT) and of LL whenever possible in the acute phase (see Table 2.2. in the Supplementary Material) [31].

#### Interventions

**Fig. 3:**
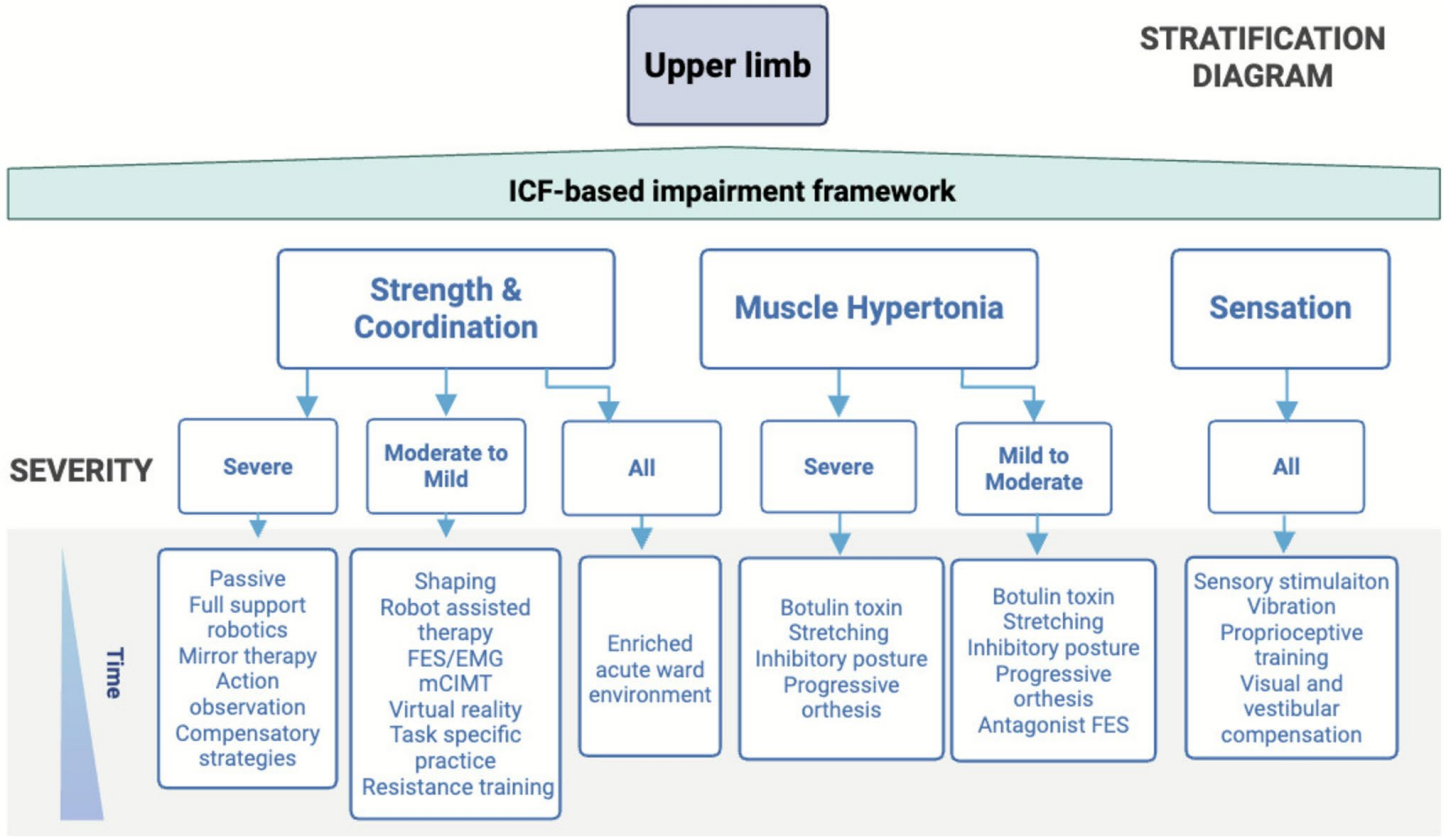
Upper limb impairment flowchart for evidence-based interventions. Treatment strategies based on different impairments: strength and coordination, muscle hypertonia, bradykinesia, tremor, and sensation

We describe treatment strategies based on six key impairments: strength and coordination, muscle hypertonia, bradykinesia, tremor, and sensation (Fig. 3). Each domain is further stratified by severity, based on the previously described clinical and instrumental assessments. For individuals with severe strength and/or coordination impairments, passive mobilization, full support robotic training, mirror therapy, action observation, and eventually compensatory strategies are recommended. The goal of these interventions is to re-activate the motor pathways first by somatosensory stimulation since the motor cortex shows sensory responses and since the motor cortex receives strong connections from primary somatosensory cortex [32]. The influence of somatosensory stimulation in rehabilitation is highlighted in this review [33]. Conversely, mirror therapy and action observation promote motor activation top-down through mental imagery [34] and mirror neuron mechanisms [35].

In cases of moderate to mild impairment, therapies requiring a more active input by the participant are recommended, such as shaping techniques, robot-assisted task specific training, functional electrical stimulation (FES), modified constraint-induced movement therapy (mCIMT), virtual reality, task-specific practice, and resistance training. A critical aspect of all these active therapies is to evaluate the level of success achieved by the patient in their execution. Principles of learning and reward emphasize that subjects shall make mistakes in their training. While early rehabilitation may emphasize errorless learning to ‘jump start’ the process [36], later most studies indicate that a higher rate of errors (around 15–30%) facilitates learning by reinforcement mechanisms that involve the delivery of dopamine [37].

All deficits, regardless of their severity, may benefit from an enriched environment [38]. In a stroke rehabilitation unit, an enriched environment can be provided by offering patients with social, cognitive, and environmental stimulation. Although much support exists in the animal literature there is a dearth of human studies addressing this potential treatment [39].

For severe muscle hypertonia, targeted interventions include botulinum toxin injections, stretching, inhibitory postures, and progressive orthosis. The same interventions are suitable for mild and moderate muscle hypertonia with the addition of antagonist FES. It is important to highlight that spasticity is characterized by hyperexcitability of the stretch reflex, arising from a reduction in supraspinal inhibitory control over spinal cord motor circuits. This loss of cortical and brainstem modulation—particularly via the corticospinal and reticulospinal pathways—leads to exaggerated reflex activity and increased muscle tone in response to passive stretch. It is extremely rare—if not impossible—for motor function to remain hidden beneath spasticity. There derive two important clinical implications. First, spasticity can be decreased by improving voluntary control hence the drive of cortex and brainstem centers onto the spinal cord. There is evidence that spasticity is inversely correlated with motor function and recovery [40]. This relationship may apply more to the upper than lower extremities. Two, spasticity treatment shall be started only if there is pain, spasms, or contractures that prevent positioning or prevent activity of daily living (e.g., washing someone armpit). Bradykinesia is managed with flexibility training, aerobic training, and balance training. Tremor may benefit from relaxation techniques. Lastly, sensation impairments should be treated with sensory stimulation, vibration, proprioceptive training, and/or visual and vestibular compensation techniques.

The sequence of interventions in our figures reflects the progression of recovery (Fig. 3). In the supplementary material we also provide a detailed descriptions of each intervention to support clinicians in their effective implementation (see Supplementary material S4_Glossary of interventions). Since this CPs are tailored to centres equipped with a wide range of technologies, we provide in the glossary alternative non-technological options for interventions to ensure the scalability of CPs.

### Lower limb and gait rehabilitation

#### Clinical assessment

As in the previous paragraph, the selection of assessments for gait &balance is a pragmatic choice based on available guidelines (Supplementary material Table S3_Clinical assessments) and adaptability to the context. Following the ICF theoretical construct, we identified the following scale for the body function domain: the Fugl-Meyer Assessment for the Lower Limb (Fugl Meyer LL) [19], the Berg Balance Scale and the Trunk Control Test [41]. For the activity domain we included the 6-Minute Walk Test, 10-Meter Walk Test, Part 3 of the Unified Parkinson’s Disease Rating Scale (UPDRS) [23], and the Timed Up and Go (TUG). For the activity and participation domain scales are the Modified Rankin Scale (mRS) [42] and the Stroke Impact Scale (SIS) [43, 44], the Functional Ambulation Classification (FAC) [45]. In contrast to UL instrumental assessment, clear evidence for LL protocols and measures is lacking. The prognostic value of LL MEPs is unclear. Similarly, kinematics measure for LL have not yet defined protocols [46] to collect meaningful clinical data.

#### Interventions

**Fig. 4:**
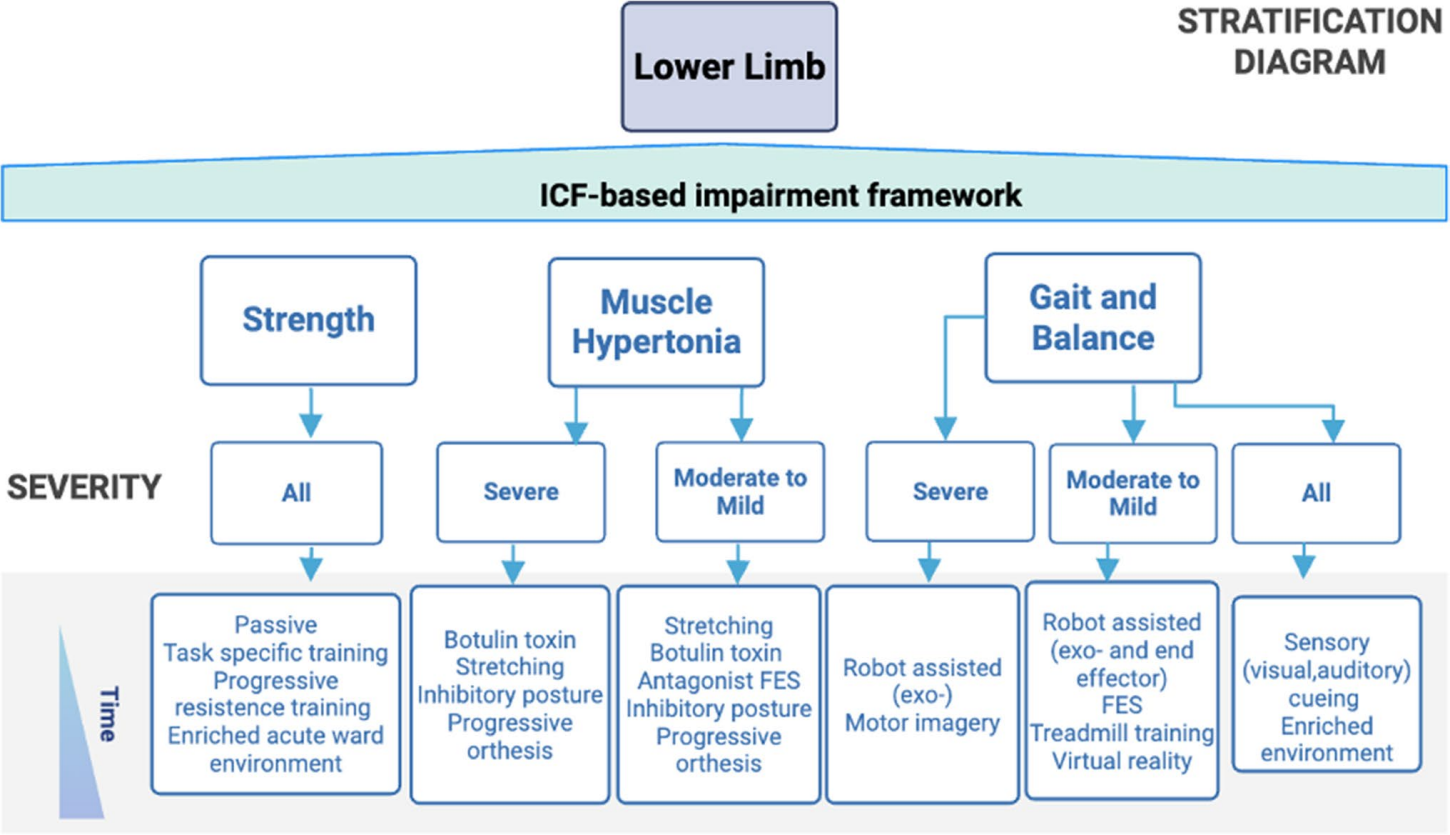
Lower limb impairment flowchart for evidence-based interventions. Treatment strategies based on different impairments: strength, muscle hypertonia, gait and balance

The stratification diagram encompasses three functional deficits: strength, muscle hypertonia, and gait & balance (Fig. 4). Each impairment is stratified by severity to guide clinicians in the selection of targeted interventions. For strength impairments all patients may benefit from passive therapy, task specific training, progressive resistance training, and enriched environment. A Cochrane review evaluating diverse physical rehabilitation approaches found that physical rehab may improve leg motor activity, balance, walking, and daily living, particularly when exceeding ~ 2.5 h/week.

Functional (task-based) training yielded greater improvements in ADLs and motor function compared to other approaches [47]. At the subacute stage several models reach high accuracy in predicting long term walking recovery [48]. The main predictive factors include trunk control, hip extension against gravity, and the FIM at 24 h yielding accuracy > 90% in prediction of walking (see the TWIST algorithm for walking prediction) [49]. For severe muscle hypertonia botulinum toxin injections, stretching routines, inhibitory postures, and the use of progressive orthosis is recommended. The use of botulinum toxin shall adhere to the same guidelines as discussed for the upper extremity. The same can be beneficial in cases of mild to moderate spasticity with the addition of antagonist FES, if available. Severe gait & balance deficits may be addressed using robot assisted therapy (including exoskeletons), as well as motor imagery. In moderate and mild cases robot assisted therapy with assistive exoskeletons or end effectors may be introduced, as well as FES/EMG, treadmill training and virtual reality. A Cochrane review assessed treadmill training ± Body Weight Support across 56 RCTs (~ 3,100 participants). People after stroke who receive treadmill training with or without body weight support are not more likely to improve their ability to walk independently (low evidence). However, treadmill training with or without body weight support may improve walking speed and walking capacity compared with people not receiving treadmill training (moderate evidence). More specifically, people after stroke who are able to walk at the start of therapy appear to benefit most from this type of intervention, but people who are not able to walk independently at therapy onset do not benefit [50]. Another Cochrane review examined electromechanical/robotic devices plus physiotherapy (~ 2,440 participants) and found an increases in the proportion of patients achieving independent walking, with earlier post-stroke intervention deriving greater benefit. For every nine people treated with a device plus physiotherapy, probably one extra person walks independently by the end of treatment [51].

Finally, all persons with gait & balance deficits should be treated in an enriched environment, introducing sensory cueing (i.e., visual and auditory). There should also be an evalution of cognitive deficits such as spatial neglect that can impact recovery of motor function including walking. Although Cochrane’s systematic review concludes that interventions targeting spatial neglect do not reliably improve walking or balance, abundant evidence shows that spatial neglect is a potent predictor of impaired postural control and walking recovery [52].

In the supplementary material we provide a description of each intervention (see Supplementary material S4_Glossary of interventions) including considerations on which intervention should be deployed based on available resources.

### Cognitive rehabilitation

#### Clinical assessment

We provide a selection of clinical assessments for severity and outcome evaluation of cognitive impairment. As in the previous paragraphs, this is a pragmatic choice based on available guidelines (Supplementary material Table S3_Clinical assessments**)** and context. For a general cognitive screen under the body function domain, we suggest tools such as the Oxford Cognitive Screen (OCS) for post stroke deficits and the Montreal Cognitive Assessment (MoCA), OR Mini-Mental State Examination (MMSE) evaluate overall cognitive processing abilities [53–55].

The OCS, MoCA, and MMSE each offer distinct advantages and limitations in screening cognitive impairment. The MoCA is considered more sensitive than the MMSE for identifying mild cognitive impairment and early dementia, as it includes tasks that better capture executive and visuospatial functions, thus providing improved assessment of right-hemisphere dysfunction [56]. However, it remains limited in evaluating domain-specific deficits common after stroke, such as apraxia or spatial neglect. In contrast, the OCS was explicitly developed for post-stroke populations and incorporates subtests for calculation, apraxia, neglect, and language, allowing for a more comprehensive evaluation of stroke-related cognitive deficits than either the MoCA or MMSE [57]. The MMSE, while widely used and useful for tracking progression of moderate to severe dementia, is less sensitive to early cognitive decline, provides minimal coverage of right-hemisphere or executive functions, and fails to assess neglect and apraxia.

On the other side, to characterize in depth the cognitive profile of each patient, specific cognitive tests with normative validation in the Italian population should be considered. Regarding spatial neglect, for example, the Behavioral Inattention Test (BIT) [58] is the most widespread battery used to assess perceptual-attentional deficits and can be categorized under body function. The Catherine Bergego Scale (CBS), [59] on the other hand, represents the most widely used scale to evaluate the impact of neglect in everyday life functionality, covering also the other ICF domains activity and participation. Similarly, all individual cognitive domains can be explored in depth with standardized tests, that measure the severity of deficit in every specific functions. In Table S3, the most important tests divided for the main cognitive domains (spatial neglect, auditory and visual attention, memory, executive function) are reported.

Encompassing all ICF domains, the Unified Parkinson’s Disease Rating Scale (UPDRS) and the SIS [43, 60] evaluate the effects of sustained attention deficits across multiple domains of the ICF: body function, activity, and participation. Similarly, for memory impairments and executive functions, both the UPDRS and SIS can be used. In this context the SIS better reflects their real-life impact on daily functioning and social participation. The choice is in this case influenced on the underlying etiology of the disorder (i.e. stroke for the SIS; Parkinson’s disease for the UPDRS). In all cases, tools that cover all ICF functional domains are recommended.

Finally, no instrumental diagnostics is currently recommended for the stratification of severity of cognitive deficits. This remains a fundamental objective for future studies in clinical neurology and neuroscience.

#### Interventions

**Fig. 5:**
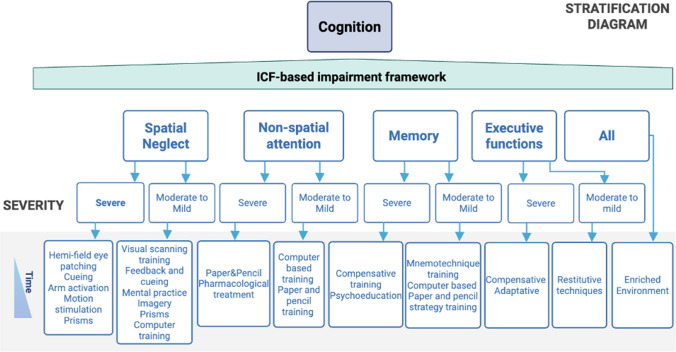
Cognitive impairment flowchart for evidence-based interventions. Treatment strategies based on different impairments: spatial neglect, non-spatial attention, memory, executive functions

The stratification diagram encompasses four functional deficits: spatial neglect, non-spatial attention, memory, and executive functions impairment (Fig. 5). Each impairment is stratified by severity. For spatial neglect, interventions range from bottom-up strategies like hemi-field eye patching, cueing, and motion stimulation for severe cases, to more cognitively demanding techniques such as visual scanning training, mental imagery, and computer-based exercises (such as go-no-go, visual search exercises) for milder presentations [52]. Prisms adaptation therapy can be used in both cases [61, 62].

It is important to highlight that the evidence for efficacy of non-pharmacological intervention in neglect is scant [52]. Longley et al. concluded that no non-pharmacological rehabilitation intervention reliably improves functional independence, balance, mobility, or walking outcomes in patients with spatial neglect. Although some methods—including prism adaptation and body-awareness treatments—may produce short-term gains on neglect assessments, there is no robust evidence of sustained functional benefit.”

Pharmacological agents for severe deficits (including methylphenidate, rivastigmine and amantadine) [52, 63–65] should be considered to modulate alertness. However, a Cochrane review assessing pharmacological interventions such as cholinergic, dopaminergic, and noradrenergic agents for treating spatial neglect concluded that the effectiveness and safety of pharmacological interventions for unilateral spatial neglect are uncertain. Larger, well-designed RCTs are needed. [64]

Moderate to mild deficits benefit from more interactive, computer-based cognitive training, including for example the tonic alertness training (i.e., TAPAT) [66]. This RCT reported yielded a large reduction in spatial bias and modest but statistically significant improvements in functional ability. These gains persisted at 3-month follow-up, supporting the potential real-world value of alertness-based digital therapies in neglect.

Memory rehabilitation includes both compensatory and restitutive strategies, according to the severity of impairment. In the first group, external memory aids and psycho-education represent the most effective instruments, while milder deficits can be treated by using restitutive techniques, that require higher cognitive resources. Some example are mnemotechnic training and structured cognitive retraining with digital or paper formats. These include for example errorless learning, vanishing cues, and spaced retrieval training. Cochrane review evidence (CD002293) [67] shows that cognitive rehabilitation yields small-to-moderate immediate reductions in self-reported memory deficits post-stroke, but these gains are not sustained over time and do not translate into functional improvements in independence or quality of life. Higher-level guidelines—for both stroke and moderate-to-severe brain injury—recommend strategic memory training (e.g., visual imagery, association, diaries, electronic cues) as a practice standard for mild-to-moderate deficit, with external compensation strategies strongly advised for functional everyday performance. Though computer-assisted working memory interventions show promise for cognitive gains, evidence of their effect on real-world functioning remains underdeveloped.

For executive function deficits, severe impairments are approached with compensatory and adaptive techniques like self-instruction, video feedback, and the use of diaries, while restitutive strategies such as goal management training and behavioral regulation are suited for milder deficits. However, one shall be mindful that Cochrane level evidence is missing. A Cochrane review (CD008391, 2010) examined 19 RCTs (907 participants) comparing cognitive rehabilitation interventions (restorative or compensatory) against standard care, no treatment or placebo. It found no convincing evidence that such interventions improve global executive function, its component domains, activities of daily living (ADLs), quality of life, or vocational outcome. More recently there has been moderate evidence for Goal Management Training having significant positive effects on executive functioning (g = 0.227), working memory (g = 0.438), performance on instrumental activities of daily living (g = 0.390), mental health (g = 0.309), and long-term memory (g = 0.269) in individuals with executive function deficits [68]. Lastly, an enriched environment is recommended across all cognitive domains as a general approach to stimulate cognitive engagement and support recovery [69, 70] with the caveat noted above about the scarce evidence in humans.

In the supplementary material we provide a detailed description of each intervention (see Supplementary material S4_Glossary of interventions).

### Speech and language rehabilitation

#### Clinical assessment

We provide a selection of clinical assessments for severity and outcome evaluation of speech and language impairment. As in the previous paragraphs, this is a pragmatic choice based on available guidelines (Supplementary material Table S3_Clinical assessments) and context. Initial screening tools such as the ELLM and SAND focus on identifying impairments at the body function level, providing a preliminary overview of speech and language deficits. More in-depth tools, including the AAT, ENPA, and BADA, are employed to evaluate specific language functions such as comprehension, production, phonological processing, and lexical access. Additionally, the “*Profilo di valutazione della disartria*” specifically addresses motor speech components for dysarthria in an Italian context [71] and has been adapted from the Robertson dysarthria profile test [72]. To capture the broader impact of aphasia on a person's life, communication-focused assessments such as APACS span all ICF domains—body function, activity, and participation—while tools like COAST, I-ASHA-FACS, and SIS further assess the functional and social implications of aphasia.

As for cognitive deficits, currently no instrumental diagnostics is recommended for the stratification of severity of speech and language deficits.

#### Interventions

**Fig. 6:**
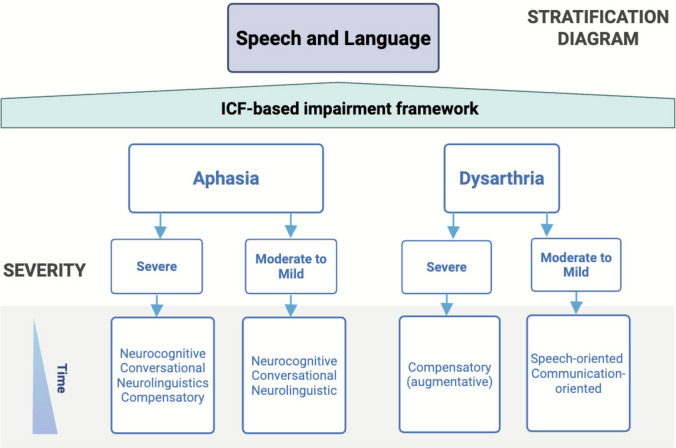
Speech and language impairment flowchart for evidence-based interventions. Treatment strategies based on aphasia and dysarthria deficits for acute and chronic neurological disorders

The stratification diagram encompasses two functional deficits: aphasia and dysarthria (Fig. 6). These deficits are further classified based on the underlying neurological disorder, differentiating acute versus neurodegenerative/progressive disorders. This choice is driven by the fact that the temporal criterion of interventions may vary based on etiology. Finally, each impairment is stratified by severity. In cases of severe aphasia, e.g. post-stroke or TBI, evidence-based strategies including speech production, phonomotor treatment, elaboration techniques, and semantic feature analysis aim to rebuild core language processing skills. Conversational and compensatory approaches—such as PACE (i.e. Promoting Aphasics' Communication Effectiveness) and multimodality aphasia therapy—are also used to enhance functional communication. For moderate to mild aphasia, similar linguistic and cognitive-linguistic treatments are employed, with emphasis on higher-level language tasks and conversational engagement. For example, Melodic intonation therapy (MIT), which uses melodic and rhythmic elements of speech to engage right-hemisphere language pathways, has shown effectiveness in improving expressive language abilities in individuals with non-fluent aphasia [73–75].

In progressive neurological conditions, severe cases may benefit from compensatory (i.e. augmentative) techniques, while moderate to mild aphasia may be supported through conversational and neurocognitive training (i.e. speech production, phonomotor treatment, elaboration techniques, semantic feature analysis) to prolong functional communication.

Cochrane level evidence exists for the efficacy of speech and language training [76]. Specifically, based on 27 randomized controlled trials including 1,620 stroke survivors with aphasia individuals receiving speech and language therapy showed significant improvements in functional communication (everyday speaking and interaction), receptive language (listening and reading comprehension), expressive language (speaking and writing). Interesting high-intensity or high-dose SLT (more frequent or prolonged sessions—typically 8–10 h/week) is probably more effective than low-intensity therapy. Also, there is no clear advantage for individual vs. group therapy. Finally, studies showed no consistent advantage of computer-delivered therapy over professional-delivered therapy.

An important issue in speech and language rehabilitation is whether therapy shall be focused on specific impairments (e.g., phonetic deficits), as for instance described in care paths based on a specific assessment of psycholinguistic skills (e.g., Fucetola et al.) [77], or whether it should be targeting in parallel multiple domains of language under the assumptions that aphasics do not have a single impairment but typically show correlated deficits across multiple functions (e.g., comprehension, reading, conversation) [78].

For dysarthria, the classification distinguishes between compensatory strategies like augmentative communication for severe motor speech deficits and speech- or communication-oriented training for milder forms (e.g., the Lee Silverman voice treatment) [79]. These interventions aim to either restore articulatory precision or optimize communication effectiveness through both direct and adaptive techniques. As in the previous paragraphs, in the supplementary material we provide a description of each intervention (see Supplementary material S4_Glossary of interventions).

### Dysphagia rehabilitation

#### Assessment

Dysphagia assessment involves an integrated clinical and instrumental approach aimed at evaluating swallowing function comprehensively, in alignment with the ICF (International Classification of Functioning, Disability and Health) framework. Clinical or functional assessments—such as the Dysphagia Outcome and Severity Scale (DOSS), Functional Oral Intake Scale (FOIS), and Swallowing Rating Scale (SRS)—focus on the observable and experienced impairments in swallowing performance, capturing body function elements like the degree of impairment and the patient's level of oral intake. These tools provide valuable insight into how swallowing difficulties impact the individual's daily functioning and nutrition. Complementing these are instrumental assessments, like the Penetration-Aspiration Scale (PAS), which offer objective, imaging-based evaluations—typically through videofluoroscopic or fiberoptic endoscopic studies—to assess physiological aspects of swallowing, such as airway protection and bolus transit.

While swallowing evaluations should be adapted to the neurological condition and the underlying pathophysiological mechanisms of swallowing, here we represent a concise, representative selection of clinical assessments for the severity and outcome evaluation of dysphagia. As in the previous paragraphs, this is a pragmatic choice based on available guidelines (Supplementary material Table S3_Clinical assessments) and context.

Regarding screening tools for dysphagia, Cochrane evaluated 37 different bedside tools used within 48 h post-stroke to detect aspiration risk (Cochrane Review CD012679, 2021) [80]. No single tool demonstrated both high sensitivity and specificity across studies. The Bedside Aspiration Test, Gugging Swallowing Screen, and Toronto Bedside Swallowing Screening Test showed the best performance in limited single-study data and only for post-stroke dysphagia. Overall study quality was low with risk of bias and stronger validation is needed. A recent multinational consensus on dysphagia in Parkinson’s disease identified the swallowing disturbance questionnaire (SDQ) as the most appropriate self-reported patient test for these patients. They additionally considered the Munich Dysphagia test-Parkinson’s disease (MDT-PD), Swallowing Clinical Assessment Score in Parkinson’s disease (SCAS-PD) and Radboud Oral Motor Inventory for Parkinson’s disease (ROMP) as valid questionnaire-based tools for dysphagia screening in PD [81].

#### Interventions

**Fig. 7:**
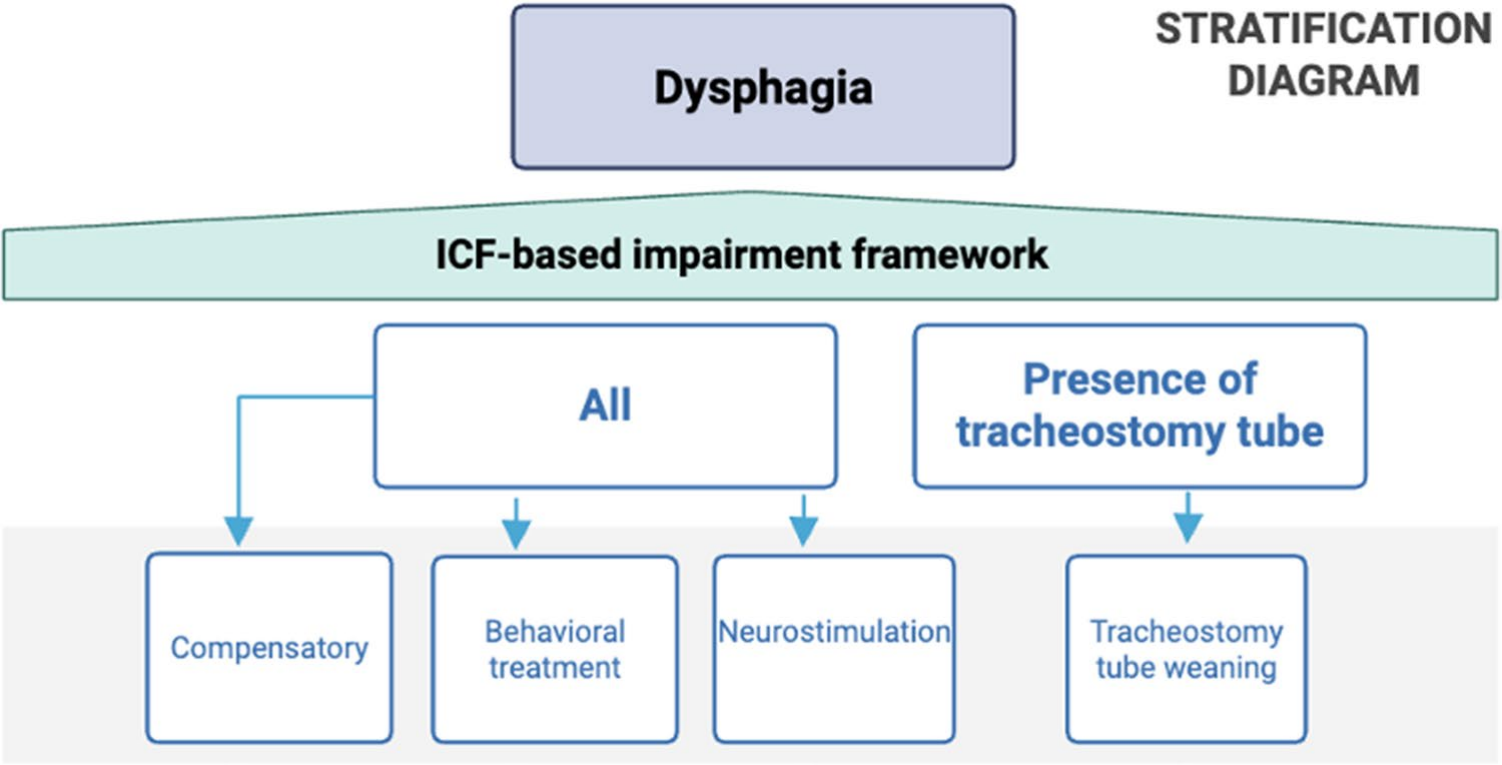
Dysphagia impairment flowchart for evidence-based interventions. Different treatment strategies are recommended for all levels of severity

The stratification diagram outlines interventions for managing dysphagia across all levels of severity (Fig. 7) [82–84]. Compensatory strategies, including postural adjustments, dietary modifications, and patient counselling, are designed to enhance swallowing safety and efficiency without altering the underlying physiology—making them suitable for immediate management across all dysphagia levels. In contrast, behavioral treatments involve rehabilitative swallowing exercises aimed at improving the strength and coordination of the swallowing mechanism through structured, repetitive tasks.

Neurostimulation techniques, such as TMS, tDCS, neuromuscular electrical stimulation (NMES), and pharyngeal stimulation, offer an adjunctive approach to promote neuroplasticity and recovery of function, and can be used with or without biofeedback [85]. These interventions, while promising, are hardly applicable in most clinical contexts due to high deployment costs, regulatory requirements, and highly trained personnel. For patients with a tracheostomy, interventions should always include a structured weaning process to support airway restoration and improve swallowing outcomes [86, 87]. As in the previous paragraphs, in the supplementary material we provide a detailed description of each intervention (see Supplementary material S4_Glossary of interventions).

The Cochrane Review CD000323 includes 41 randomized controlled trials involving approximately 2,660 stroke patients, assessing a range of interventions: behavioural therapy, acupuncture, drug treatments, NMES, PES, physical stimulation, tDCS, and rTMS. There was no significant impact on death or dependency (moderate-quality evidence). There was a likely shorter hospital stay (mean reduction ~ 2.9 days; moderate-quality evidence), fewer chest infections, and fewer persistent cases of dysphagia (low to very low-quality evidence). Therapy may improve swallowing abilities, but evidence quality is very low and heterogeneity substantial. No clear evidence of reduced aspiration scores emerged [88].

A recent consensus on the treatment of dysphagia in Parkinson’s disease suggest standard swallowing therapy (i.e. compensatory and behavioral treatment) for patients with a sufficient cognitive level [89]. No rTMS, tDCS or NMES are currently recommended for this population. Among pharmacological options, botulinum toxin (BT) may be considered for the treatment of dysphagia due to upper esophageal sphincter (UES) dysfunction [90].

Overall, multiple modalities show promise, but conclusions are limited by variable-quality trials and inconsistent outcome definitions. Most benefits appear in reducing complications rather than overall dependency.

## Conclusion

These clinical practice guidelines aim to provide an evidence-based, pragmatic pathway to ensure the highest standards of neurorehabilitation within a hospital setting. The indications encompass all impairments, including motor, cognitive, speech and language and dysphagia deficits, adopting a behavioral based classification instead of an etiological one. A comprehensive review of existing rehabilitation guidelines provided the foundational basis for the expert group to draw upon their clinical expertise and design pathways that integrate evidence-based interventions within the local context. Indeed, the final output adheres to the recommendations of the major international guidelines. The schematic and user-friendly tables and flow charts offer clinicians an immediate reference guide to implement the optimal approach based on the type and severity of impairment along a continuous spectrum, which can be followed iteratively according to the individual patient's progress. Although specific assessment cutoff values were not provided to dictate the temporal sequence of interventions, this schematic approach facilitates the establishment of a consistent operational methodology among all clinicians involved. While accommodating individual patient progression these flowcharts are intended for the acute/subacute phase of acute neurological disorders and chronic neurodegenerative pathologies.

To ensure proper adoption of this working methodology and to promote a shared working culture, the expert group engaged all disciplines of the multidisciplinary team. Integral components to ensure the transferability of the pathway include educational training initiatives, the identification of group leaders and the setting domain, as well as regular reassessments of activities [91].

The definition of Clinical Pathways also addresses the necessity to standardize interventions from a research perspective, mitigating confounding factors associated with varying treatments and protocols to which patients are subjected. Toward this objective, the adoption of this approach by many rehabilitative facilities, may facilitate the creation of a Regional/National neurorehabilitation Registry.

We acknowledge that a potential limitation to implementation lies in the availability of technologies. The main technologies included in our guidelines are robotics, virtual reality and neurostimulation for dysphagia treatment. Sound evidence exists only for robotic gait training, which seems to be effective in the subacute phase after stroke for regaining independent ambulation [92]. In fact, evidence from a recent meta-analysis questions the clinical transferability of upper limb robotic training [93]. Nonetheless, incorporating robotic training alongside other techniques may augment the intensity and dosage of rehabilitation for UL [94]. Although the cost of robotic devices for gait training is becoming less restrictive, this technology in particular remains the main limitation in low resource settings. In these cases, gait training shall be provided with conventional therapy by two therapists, with a particular focus on alternating LL patterns. Virtual reality, on the other hand, is a scalable and potentially low-cost technology that may be available in most middle to high-income healthcare facilities. Neurostimulation, on the contrary, remains rarely accessible and is, for the time being, relegated to research settings in Italy. Future work will need to provide consistent scientific designs in, possibly, large multi-center collaborations to effectively increase the application of neuromodulation. In addition, our work shows instrumental evaluations in the field seem to provide useful information only in the motor domain for upper and lower limb function (i.e. the MEP). While many biomarkers have been identified for other domains of function (i.e. cognitive and speech and language deficits), these features do not effectively stratify interventions, and for the time being clinical pathways rely entirely on clinical and/or neuropsychological evaluations (see above). In this context, additional stratification metrics based on instrumental diagnostics may be pivotal to effectively apply neurostimulation or other interventions in a neurorehabilitation setting. This remains a fundamental objective for future studies in clinical neurology and neuroscience.

Finally, it is important to emphasize that technology is not a crucial factor for implementation. Instead, technology should be integrated as a complementary component to therapist-guided interventions, which may also serve as a contingency measure in the absence of technological resources.

The present work did not address the emerging topic within the scientific community regarding the optimal dose and intensity of training. This decision was guided by the practical constraints imposed by the Italian national health system, which sees a pragmatic limit of "intensive" treatment due to limited resources (i.e. 3 h per day).

With a focus on evidence-based rehabilitation, these guidelines aim to ensure the highest standards of neurorehabilitation care. Successful wide-scale implementation across regional/national settings will require the identification of barriers and facilitators, examining organizational readiness, and developing strategies to maximize adoption fidelity. In this context, an interdisciplinary approach will be crucial for realizing these future perspectives.

## Supplementary Information

Below is the link to the electronic supplementary material.

Supplementary file1 (DOCX 109 KB)

Supplementary file2 (DOCX 118 KB)

## Declarations

### Data availability

All data reported in the present study are available from the authors upon reasonable request.
