# Supplementary material for: Neurorehabilitation Clinical Pathways in Stroke: The Padova Model
Source: Neurol Sci. 2026 Feb 13;47(3):256. doi: 10.1007/s10072-025-08770-y (PMC12901142; doi:10.1007/s10072-025-08770-y)
Supplement: Supplementary file 2 — Supplementary file2 (DOCX 118 KB) [file 10072_2025_8770_MOESM2_ESM.docx]

**Supplementary material**

**S1. Query strings**

**Stroke:** (((((adult) AND (human)) AND (stroke)) ) AND (recovery of function)) OR (stroke rehabilitation) Filters: Guideline

**Speech and Language:** ((((stroke/therapy)) AND (aphasia/therapy)) AND (aphasia/etiology)) AND (stroke/complications) Filters: Guideline; ((((stroke/therapy)) AND (aphasia/therapy)) AND (aphasia/etiology)) AND (stroke/complications) Filters: Review, Systematic Review, Guideline

**Cognition:** ((brain disease) OR (cognition disorder)) AND (rehabiitation) - Spellcheck off Filters: Guideline

**Dysphagia:** ((dysphagia) OR (swallowing disorder)) AND (rehabiitation) - Spellcheck off Filters: Guideline

**S2. List of considered Neurorehabilitation guidelines**

1: Kjellström T, Norrving B, Shatchkute A. Helsingborg Declaration 2006 on European stroke strategies. Cerebrovasc Dis. 2007;23(2-3):231-41. doi: 10.1159/000097646. Epub 2006 Nov 30. PMID: 17139166.

2: Stroke rehabilitation in adults - NICE guideline [NG236] Published: 18 October 2023 (<https://www.nice.org.uk/guidance/ng236>)

~~3: Osborne JA, Botkin R, Colon-Semenza C, DeAngelis TR, Gallardo OG, Kosakowski H, Martello J, Pradhan S, Rafferty M, Readinger JL, Whitt AL, Ellis TD. Physical Therapist Management of Parkinson Disease: A Clinical Practice Guideline From the American Physical Therapy Association. Phys Ther. 2022 Apr 1;102(4):pzab302. doi: 10.1093/ptj/pzab302. Erratum in: Phys Ther. 2022 Aug 1;102(8):pzac098. doi: 10.1093/ptj/pzac098. PMID: 34963139; PMCID: PMC9046970.~~

~~4: Grimes D, Fitzpatrick M, Gordon J, Miyasaki J, Fon EA, Schlossmacher M, Suchowersky O, Rajput A, Lafontaine AL, Mestre T, Appel-Cresswell S, Kalia SK, Schoffer K, Zurowski M, Postuma RB, Udow S, Fox S, Barbeau P, Hutton B. Canadian guideline for Parkinson disease. CMAJ. 2019 Sep 9;191(36):E989-E1004. doi: 10.1503/cmaj.181504. PMID: 31501181; PMCID: PMC6733687.~~

~~5: Norlin JM, Willis M, Persson U, Andersson E, E Pålhagen S, Odin P. Swedish guidelines for device-aided therapies in Parkinson's disease -Economic evaluation and implementation. Acta Neurol Scand. 2021 Aug;144(2):170-178. doi: 10.1111/ane.13434. Epub 2021 Apr 26. PMID: 33899213.~~

~~6.Keus, S., Munneke, M., Graziano, M., et al. (2014) European Physiotherapy Guideline for Parkinson’s Disease. The Netherlands: KNGF/ParkinsonNet, 1-191.~~

3: Winstein CJ, Stein J, Arena R, Bates B, Cherney LR, Cramer SC, Deruyter F,Eng JJ, Fisher B, Harvey RL, Lang CE, MacKay-Lyons M, Ottenbacher KJ, Pugh S,Reeves MJ, Richards LG, Stiers W, Zorowitz RD; American Heart Association Stroke Council, Council on Cardiovascular and Stroke Nursing, Council on Clinical Cardiology, and Council on Quality of Care and Outcomes Research. Guidelines for Adult Stroke Rehabilitation and Recovery: A Guideline for Healthcare Professionals From the American Heart Association/American Stroke Association. Stroke. 2016 Jun;47(6):e98-e169. doi: 10.1161/STR.0000000000000098. Epub 2016 May 4. Erratum in: Stroke. 2017 Feb;48(2):e78. doi: 10.1161/STR.0000000000000120. Erratum in: Stroke. 2017 Dec;48(12):e369. doi: 10.1161/STR.0000000000000156. PMID: 27145936.

4: Minelli C, Luvizutto GJ, Cacho RO, Neves LO, Magalhães SCSA, Pedatella MTA, Mendonça LIZ, Ortiz KZ, Lange MC, Ribeiro PW, Souza LAPS, Milani C, Cruz DMCD, Costa RDMD, Conforto AB, Carvalho FMM, Ciarlini BS, Frota NAF, Almeida KJ, Schochat E, Oliveira TP, Miranda C, Piemonte MEP, Lopes LCG, Lopes CG, Tosin MHS, Oliveira BC, Oliveira BGRB, Castro SS, Andrade JBC, Silva GS, Pontes-Neto OM, Carvalho JJF, Martins SCO, Bazan R. Brazilian practice guidelines for stroke rehabilitation: Part II. Arq Neuropsiquiatr. 2022 Jul;80(7):741-758. doi: 10.1055/s-0042-1757692. Epub 2022 Sep 29. PMID: 36254447; PMCID: PMC9685826.

5: Hebert D, Lindsay MP, McIntyre A, Kirton A, Rumney PG, Bagg S, Bayley M, Dowlatshahi D, Dukelow S, Garnhum M, Glasser E, Halabi ML, Kang E, MacKay-Lyons M, Martino R, Rochette A, Rowe S, Salbach N, Semenko B, Stack B, Swinton L, Weber V, Mayer M, Verrilli S, DeVeber G, Andersen J, Barlow K, Cassidy C, Dilenge ME, Fehlings D, Hung R, Iruthayarajah J, Lenz L, Majnemer A, Purtzki J, Rafay M, Sonnenberg LK, Townley A, Janzen S, Foley N, Teasell R. Canadian stroke best practice recommendations: Stroke rehabilitation practice guidelines, update 2015. Int J Stroke. 2016 Jun;11(4):459-84. doi: 10.1177/1747493016643553. Epub 2016 Apr 14. PMID: 27079654.

6: Zhang T, Zhao J, Li X, Bai Y, Wang B, Qu Y, Li B, Zhao S; Chinese Stroke Association Stroke Council Guideline Writing Committee. Chinese Stroke Association guidelines for clinical management of cerebrovascular disorders: executive summary and 2019 update of clinical management of stroke rehabilitation. Stroke Vasc Neurol. 2020 Sep;5(3):250-259. doi: 10.1136/svn-2019-000321. Epub 2020 Jun 28. PMID: 32595138; PMCID: PMC7548515.

7: MacKay-Lyons M, Billinger SA, Eng JJ, Dromerick A, Giacomantonio N, Hafer- Macko C, Macko R, Nguyen E, Prior P, Suskin N, Tang A, Thornton M, Unsworth K. Aerobic Exercise Recommendations to Optimize Best Practices in Care After Stroke: AEROBICS 2019 Update. Phys Ther. 2020 Jan 23;100(1):149-156. doi: 10.1093/ptj/pzz153. PMID: 31596465; PMCID: PMC8204880.

8: Tang E, Moran N, Cadman M, Hill S, Sloan C, Warburton E; guideline committee. Stroke rehabilitation in adults: summary of updated NICE guidance. BMJ. 2024 Mar 22;384:q498. doi: 10.1136/bmj.q498. PMID: 38519084.

9: Stein J, Katz DI, Black Schaffer RM, Cramer SC, Deutsch AF, Harvey RL, Lang CE, Ottenbacher KJ, Prvu-Bettger J, Roth EJ, Tirschwell DL, Wittenberg GF, Wolf SL, Nedungadi TP. Clinical Performance Measures for Stroke Rehabilitation: Performance Measures From the American Heart Association/American Stroke Association. Stroke. 2021 Oct;52(10):e675-e700. doi: 11.1161/STR.0000000000000388. Epub 2021 Aug 5. PMID: 34348470.

10: Cameron JI, O'Connell C, Foley N, Salter K, Booth R, Boyle R, Cheung D, Cooper N, Corriveau H, Dowlatshahi D, Dulude A, Flaherty P, Glasser E, Gubitz G, Hebert D, Holzmann J, Hurteau P, Lamy E, LeClaire S, McMillan T, Murray J, Scarfone D, Smith EE, Shum V, Taylor K, Taylor T, Yanchula C, Teasell R, Lindsay P; Heart and Stroke Foundation Canadian Stroke Best Practice Committees. Canadian Stroke Best Practice Recommendations: Managing transitions of care following Stroke, Guidelines Update 2016. Int J Stroke. 2016 Oct;11(7):807-22. doi: 12.1177/1747493016660102. Epub 2016 Jul 21. PMID: 27443991.

11: Blacquiere D, Lindsay MP, Foley N, Taralson C, Alcock S, Balg C, Bhogal S, Cole J, Eustace M, Gallagher P, Ghanem A, Hoechsmann A, Hunter G, Khan K, Marrero A, Moses B, Rayner K, Samis A, Smitko E, Vibe M, Gubitz G, Dowlatshahi D, Phillips S, Silver FL; Heart and Stroke Foundation Canadian Stroke Best Practice Committees. Canadian Stroke Best Practice Recommendations: Telestroke Best Practice Guidelines Update 2017. Int J Stroke. 2017 Oct;12(8):886-895. doi: 1.1177/1747493017706239. Epub 2017 Apr 26. PMID: 28441928.

12: Miller EL, Murray L, Richards L, Zorowitz RD, Bakas T, Clark P, Billinger SA; American Heart Association Council on Cardiovascular Nursing and the Stroke Council. Comprehensive overview of nursing and interdisciplinary rehabilitation care of the stroke patient: a scientific statement from the American Heart Association. Stroke. 2010 Oct;41(10):2402-48. doi: 10.1161/STR.0b013e3181e7512b. Epub 2010 Sep 2. PMID: 20813995.

13: KNGF Practice Guideline Stroke Rehabilitation, J.M. Verbeek, , E.E.H. van Wegen, R.P.S. van Peppen , H.J.M. Hendriks, M.B. Rietberg, Ph.J. van der Wees H, K. Heijblom, A.A.G. Goos, W.O. HanssenI, B.C. Harmeling-van der We, L.D. de Jong, J.F. KamphuisXII, M.M. Noom, R. van der Schaft, C.J. Smeets, T.P.M.M. Vluggen, D.R.B. Vijsma, C.M. Vollmar, G. Kwakkel <https://www.dsnr.nl/wp-content/uploads/2012/03/stroke_practice_guidelines_2014.pdf>

14: Clinical Pathways in Stroke Rehabilitation Evidence-based Clinical Practice Recommendations. Platz 2021. ISBN 978-3-030-58504-4 ISBN 978-3-030-58505-1 (eBook) <https://doi.org/10.1007/978-3-030-58505-1>

15: Teasell R, Salbach NM, Foley N, Mountain A, Cameron JI, Jong A, Acerra NE, Bastasi D, Carter SL, Fung J, Halabi ML, Iruthayarajah J, Harris J, Kim E, Noland A, Pooyania S, Rochette A, Stack BD, Symcox E, Timpson D, Varghese S, Verrilli S, Gubitz G, Casaubon LK, Dowlatshahi D, Lindsay MP. Canadian Stroke Best Practice Recommendations: Rehabilitation, Recovery, and Community Participation following Stroke. Part One: Rehabilitation and Recovery Following Stroke; 6th Edition Update 2019. Int J Stroke. 2020 Oct;15(7):763-788. doi: 10.1177/1747493019897843. Epub 2020 Jan 27. PMID: 31983296.

16: Vitti E, Hillis AE. Treatment of post-stroke aphasia: A narrative review for stroke neurologists. Int J Stroke. 2021 Dec;16(9):1002-1008. doi: 10.1177/17474930211017807. Epub 2021 Jun 6. PMID: 33949274; PMCID: PMC8645656.

17: Mattioli F. The clinical management and rehabilitation of post stroke aphasia in Italy: evidences from the literature and clinical experience. Neurol Sci. 2019 Jul;40(7):1329-1334. doi: 10.1007/s10072-019-03844-0. Epub 2019 Mar 21. PMID: 30900098.

18: Pérez-Martínez V, Zorzo C, Méndez M. Differential approach to stroke aphasia and primary progressive aphasia using transcranial magnetic stimulation: A systematic review. Acta Neurobiol Exp (Wars). 2023 Sep 29;83(3):280-298. doi: 10.55782/ane-2023-2433. PMID: 37874189.

19: Saxena S, Hillis AE. An update on medications and noninvasive brain stimulation to augment language rehabilitation in post-stroke aphasia. Expert Rev Neurother. 2017 Nov;17(11):1091-1107. doi: 10.1080/14737175.2017.1373020. Epub 2017 Sep 8. PMID: 28847186.

20: Ntasiopoulou C, Nasios G, Messinis L, Nousia A, Siokas V, Dardiotis E. Repetitive Transcranial Magnetic Stimulation in Post-stroke Aphasia: Comparative Evaluation of Inhibitory and Excitatory Therapeutic Protocols: Narrative Review. Adv Exp Med Biol. 2023;1425:619-628. doi: 10.1007/978-3-031-31986-0_60. PMID: 37581835.

21: Kielar A, Patterson D, Chou YH. Efficacy of repetitive transcranial magnetic stimulation in treating stroke aphasia: Systematic review and meta-analysis. Clin Neurophysiol. 2022 Aug;140:196-227. doi: 10.1016/j.clinph.2022.04.017. Epub 2022 May 5. PMID: 35606322.

22: Sebastian R, Tsapkini K, Tippett DC. Transcranial direct current stimulation in post stroke aphasia and primary progressive aphasia: Current knowledge and future clinical applications. NeuroRehabilitation. 2016 Jun 13;39(1):141-52. doi: 10.3233/NRE-161346. PMID: 27314871; PMCID: PMC4964590.

23: Pierce JE, Menahemi-Falkov M, O'Halloran R, Togher L, Rose ML. Constraint and multimodal approaches to therapy for chronic aphasia: A systematic review and meta-analysis. Neuropsychol Rehabil. 2019 Aug;29(7):1005-1041. doi: 10.1080/09602011.2017.1365730. Epub 2017 Sep 18. PMID: 28920522.

24: Ehsaan F, Mumtaz N, Saqulain G. Novel therapeutic techniques for post stroke aphasia: A narrative review. J Pak Med Assoc. 2022 Jan;72(1):121-125. doi: 10.47391/JPMA.2277. PMID: 35099450.

25: Han C, Tang J, Tang B, Han T, Pan J, Wang N. The effectiveness and safety of noninvasive brain stimulation technology combined with speech training on aphasia after stroke: A systematic review and meta-analysis. Medicine (Baltimore). 2024 Jan 12;103(2):e36880. doi: 10.1097/MD.0000000000036880. PMID: 38215135; PMCID: PMC10783273.

26: Zhang J, Zhong D, Xiao X, Yuan L, Li Y, Zheng Y, Li J, Liu T, Jin R. Effects of repetitive transcranial magnetic stimulation (rTMS) on aphasia in stroke patients: A systematic review and meta-analysis. Clin Rehabil. 2021 Aug;35(8):1103-1116. doi: 10.1177/0269215521999554. Epub 2021 Mar 11. PMID: 33706572.

27: Tilton-Bolowsky VE, Hillis AE. A Review of Poststroke Aphasia Recovery and Treatment Options. Phys Med Rehabil Clin N Am. 2024 May;35(2):419-431. doi: 10.1016/j.pmr.2023.06.010. Epub 2023 Jul 11. PMID: 38514227.

28: Bucur M, Papagno C. Are transcranial brain stimulation effects long-lasting in post-stroke aphasia? A comparative systematic review and meta-analysis on naming performance. Neurosci Biobehav Rev. 2019 Jul;102:264-289. doi: 10.1016/j.neubiorev.2019.04.019. Epub 2019 May 8. PMID: 31077693.

29: Gholami M, Pourbaghi N, Taghvatalab S. Evaluation of rTMS in patients with poststroke aphasia: a systematic review and focused meta-analysis. Neurol Sci. 2022 Aug;43(8):4685-4694. doi: 10.1007/s10072-022-06092-x. Epub 2022 May 2. PMID: 35499630.

30: ALHarbi MF, Armijo-Olivo S, Kim ES. Transcranial direct current stimulation (tDCS) to improve naming ability in post-stroke aphasia: A critical review. Behav Brain Res. 2017 Aug 14;332:7-15. doi: 10.1016/j.bbr.2017.05.050. Epub 2017 May 29. PMID: 28572057.

31: Balossier A, Etard O, Descat C, Vivien D, Emery E. Epidural Cortical Stimulation as a Treatment for Poststroke Aphasia: A Systematic Review of the Literature and Underlying Neurophysiological Mechanisms. Neurorehabil Neural Repair. 2016 Feb;30(2):120-30. doi: 10.1177/1545968315606989. Epub 2015 Sep 29. PMID: 26422832.

32: Fama ME, Turkeltaub PE. Treatment of poststroke aphasia: current practice and new directions. Semin Neurol. 2014 Nov;34(5):504-13. doi: 10.1055/s-0034-1396004. Epub 2014 Dec 17. PMID: 25520022.

33: Minelli C, Luvizutto GJ, Cacho RO, Neves LO, Magalhães SCSA, Pedatella MTA, Mendonça LIZ, Ortiz KZ, Lange MC, Ribeiro PW, Souza LAPS, Milani C, Cruz DMCD, Costa RDMD, Conforto AB, Carvalho FMM, Ciarlini BS, Frota NAF, Almeida KJ, Schochat E, Oliveira TP, Miranda C, Piemonte MEP, Lopes LCG, Lopes CG, Tosin MHS, Oliveira BC, Oliveira BGRB, Castro SS, Andrade JBC, Silva GS, Pontes-Neto OM, Carvalho JJF, Martins SCO, Bazan R. Brazilian practice guidelines for stroke rehabilitation: Part II. Arq Neuropsiquiatr. 2022 Jul;80(7):741-758. doi: 10.1055/s-0042-1757692. Epub 2022 Sep 29. PMID: 36254447; PMCID: PMC9685826.

~~34: Osborne JA, Botkin R, Colon-Semenza C, DeAngelis TR, Gallardo OG, Kosakowski H, Martello J, Pradhan S, Rafferty M, Readinger JL, Whitt AL, Ellis TD. Physical Therapist Management of Parkinson Disease: A Clinical Practice Guideline From the American Physical Therapy Association. Phys Ther. 2022 Apr 1;102(4):pzab302. doi: 10.1093/ptj/pzab302. Erratum in: Phys Ther. 2022 Aug 1;102(8):pzac098. doi: 10.1093/ptj/pzac098. PMID: 34963139; PMCID: PMC9046970.~~

34: Zhang T, Zhao J, Li X, Bai Y, Wang B, Qu Y, Li B, Zhao S; Chinese Stroke Association Stroke Council Guideline Writing Committee. Chinese Stroke Association guidelines for clinical management of cerebrovascular disorders: executive summary and 2019 update of clinical management of stroke rehabilitation. Stroke Vasc Neurol. 2020 Sep;5(3):250-259. doi: 10.1136/svn-2019-000321. Epub 2020 Jun 28. PMID: 32595138; PMCID: PMC7548515.

~~36: Grimes D, Fitzpatrick M, Gordon J, Miyasaki J, Fon EA, Schlossmacher M, Suchowersky O, Rajput A, Lafontaine AL, Mestre T, Appel-Cresswell S, Kalia SK, Schoffer K, Zurowski M, Postuma RB, Udow S, Fox S, Barbeau P, Hutton B. Canadian guideline for Parkinson disease. CMAJ. 2019 Sep 9;191(36):E989-E1004. doi: 10.1503/cmaj.181504. PMID: 31501181; PMCID: PMC6733687~~.

~~37: Bayley MT, Tate R, Douglas JM, Turkstra LS, Ponsford J, Stergiou-Kita M, Kua A, Bragge P; INCOG Expert Panel. INCOG guidelines for cognitive rehabilitation following traumatic brain injury: methods and overview. J Head Trauma Rehabil. 2014 Jul-Aug;29(4):290-306. doi: 10.1097/HTR.0000000000000070. PMID: 24984093.~~

35: Giacino JT, Katz DI, Schiff ND, Whyte J, Ashman EJ, Ashwal S, Barbano R, Hammond FM, Laureys S, Ling GSF, Nakase-Richardson R, Seel RT, Yablon S, Getchius TSD, Gronseth GS, Armstrong MJ. Practice Guideline Update Recommendations Summary: Disorders of Consciousness: Report of the Guideline Development, Dissemination, and Implementation Subcommittee of the American Academy of Neurology; the American Congress of Rehabilitation Medicine; and the National Institute on Disability, Independent Living, and Rehabilitation Research. Arch Phys Med Rehabil. 2018 Sep;99(9):1699-1709. doi: 10.1016/j.apmr.2018.07.001. Epub 2018 Aug 8. PMID: 30098791.

~~36: Ponsford J, Bayley M, Wiseman-Hakes C, Togher L, Velikonja D, McIntyre A, Janzen S, Tate R; INCOG Expert Panel. INCOG recommendations for management of cognition following traumatic brain injury, part II: attention and information processing speed. J Head Trauma Rehabil. 2014 Jul-Aug;29(4):321-37. doi: 10.1097/HTR.0000000000000072. PMID: 24984095.~~

36: Blacquiere D, Lindsay MP, Foley N, Taralson C, Alcock S, Balg C, Bhogal S, Cole J, Eustace M, Gallagher P, Ghanem A, Hoechsmann A, Hunter G, Khan K, Marrero A, Moses B, Rayner K, Samis A, Smitko E, Vibe M, Gubitz G, Dowlatshahi D, Phillips S, Silver FL; Heart and Stroke Foundation Canadian Stroke Best Practice Committees. Canadian Stroke Best Practice Recommendations: Telestroke Best Practice Guidelines Update 2017. Int J Stroke. 2017 Oct;12(8):886-895. doi: 10.1177/1747493017706239. Epub 2017 Apr 26. PMID: 28441928.

~~40: Tate R, Kennedy M, Ponsford J, Douglas J, Velikonja D, Bayley M, Stergiou- Kita M. INCOG recommendations for management of cognition following traumatic brain injury, part III: executive function and self-awareness. J Head Trauma Rehabil. 2014 Jul-Aug;29(4):338-52. doi: 10.1097/HTR.0000000000000068. PMID: 24984096.~~

37: Cappa SF, Benke T, Clarke S, Rossi B, Stemmer B, van Heugten CM; Task Force on Cognitive Rehabilitation; European Federation of Neurological Societies. EFNS guidelines on cognitive rehabilitation: report of an EFNS task force. Eur J Neurol. 2005 Sep;12(9):665-80. doi: 10.1111/j.1468-1331.2005.01330.x. PMID:16128867.

~~42: Togher L, Wiseman-Hakes C, Douglas J, Stergiou-Kita M, Ponsford J, Teasell R, Bayley M, Turkstra LS; INCOG Expert Panel. INCOG recommendations for management of cognition following traumatic brain injury, part IV: cognitive communication. J Head Trauma Rehabil. 2014 Jul-Aug;29(4):353-68. doi: 10.1097/HTR.0000000000000071. PMID: 24984097.~~

~~43: Velikonja D, Tate R, Ponsford J, McIntyre A, Janzen S, Bayley M; INCOG Expert Panel. INCOG recommendations for management of cognition following traumatic brain injury, part V: memory. J Head Trauma Rehabil. 2014 Jul- Aug;29(4):369-86. doi: 10.1097/HTR.0000000000000069. PMID: 24984098.~~

~~44: Mehta S, Devito L, Patsakos EM, Devito J, Velikonja D, Bayley M, Teasell R, MacKenzie HM. Updated Canadian Clinical Practice Guideline for the Rehabilitation of Adults With Moderate to Severe Traumatic Brain Injury: Mental Health Recommendations. J Head Trauma Rehabil. 2024 Sep-Oct 01;39(5):359-368. doi: 10.1097/HTR.0000000000000961. Epub 2024 Sep 10. PMID: 39256157.~~

~~45: Ponsford J, Janzen S, McIntyre A, Bayley M, Velikonja D, Tate R; INCOG Expert Panel. INCOG recommendations for management of cognition following traumatic brain injury, part I: posttraumatic amnesia/delirium. J Head Trauma Rehabil. 2014 Jul-Aug;29(4):307-20. doi: 10.1097/HTR.0000000000000074. PMID: 24984094.~~

~~46: MacKenzie HM, Velikonja D, Devito J, Devito L, Patsakos EM, Bayley M, Teasell R, Mehta S. Updated Canadian Clinical Practice Guideline for the Rehabilitation of Adults With Moderate to Severe Traumatic Brain Injury: Behavioral Recommendations. J Head Trauma Rehabil. 2024 Sep-Oct 01;39(5):382-394. doi: 10.1097/HTR.0000000000000973. Epub 2024 Sep 10. PMID: 39256159.~~

38: Cameron JI, O'Connell C, Foley N, Salter K, Booth R, Boyle R, Cheung D, Cooper N, Corriveau H, Dowlatshahi D, Dulude A, Flaherty P, Glasser E, Gubitz G, Hebert D, Holzmann J, Hurteau P, Lamy E, LeClaire S, McMillan T, Murray J, Scarfone D, Smith EE, Shum V, Taylor K, Taylor T, Yanchula C, Teasell R, Lindsay P; Heart and Stroke Foundation Canadian Stroke Best Practice Committees. Canadian Stroke Best Practice Recommendations: Managing transitions of care following Stroke, Guidelines Update 2016. Int J Stroke. 2016 Oct;11(7):807-22. doi: 10.1177/1747493016660102. Epub 2016 Jul 21. PMID: 27443991.

~~48: Kang PB, Morrison L, Iannaccone ST, Graham RJ, Bönnemann CG, Rutkowski A, Hornyak J, Wang CH, North K, Oskoui M, Getchius TS, Cox JA, Hagen EE, Gronseth G, Griggs RC; Guideline Development Subcommittee of the American Academy of Neurology and the Practice Issues Review Panel of the American Association of Neuromuscular & Electrodiagnostic Medicine. Evidence-based guideline summary: evaluation, diagnosis, and management of congenital muscular dystrophy: Report of the Guideline Development Subcommittee of the American Academy of Neurology and the Practice Issues Review Panel of the American Association of Neuromuscular & Electrodiagnostic Medicine. Neurology. 2015 Mar 31;84(13):1369-78. doi: 10.1212/WNL.0000000000001416. PMID: 25825463; PMCID: PMC4388744.~~

39. Eapen BC, Tran J, Ballard-Hernandez J, Buelt A, Hoppes CW, Matthews C, Pundik
S, Reston J, Tchopev Z, Wayman LM, Koehn T. Stroke Rehabilitation: Synopsis of
the 2024 U.S. Department of Veterans Affairs and U.S. Department of Defense
Clinical Practice Guidelines. Ann Intern Med. 2025 Feb;178(2):249-268. doi:
10.7326/ANNALS-24-02205. Epub 2025 Jan 21. PMID: 39832369.

40. Qin H, Reid I, Gorelik A, Ng L. Environmental enrichment for stroke and other non-progressive brain injury. Cochrane Database of Systematic Reviews 2021, Issue 11. Art. No.: CD011879. DOI: 10.1002/14651858.CD011879.pub2.

41. Mehrholz J, Thomas S, Elsner B. Treadmill training and body weight support for walking after stroke. Cochrane Database of Systematic Reviews 2017, Issue 8. Art. No.: CD002840. DOI: 10.1002/14651858.CD002840.pub4.

42. Borges LRDM, Fernandes ABGS, Oliveira dos Passos J, Rego IAnanda Oliveira, Campos TF. Action observation for upper limb rehabilitation after stroke. Cochrane Database of Systematic Reviews 2022, Issue 8. Art. No.: CD011887. DOI: 10.1002/14651858.CD011887.pub3.

43. Longley V, Hazelton C, Heal C, Pollock A, Woodward-Nutt K, Mitchell C, Pobric G, Vail A, Bowen A. Non-pharmacological interventions for spatial neglect or inattention following stroke and other non-progressive brain injury. Cochrane Database of Systematic Reviews 2021, Issue 7. Art. No.: CD003586. DOI: 10.1002/14651858.CD003586.pub4.

44. Bowen A, Hazelton C, Pollock A, Lincoln NB. Cognitive rehabilitation for spatial neglect following stroke. Cochrane Database Syst Rev. 2013 Jul 1;2013(7):CD003586. doi: 10.1002/14651858.CD003586.pub3. Update in: Cochrane Database Syst Rev. 2021 Jul 1;7:CD003586. doi: 10.1002/14651858.CD003586.pub4. PMID:

~~55. Hoare BJ, Wallen MA, Imms C, Villanueva E, Rawicki HB, Carey L. Botulinum toxin A as an adjunct to treatment in the management of the upper limb in children with spastic cerebral palsy (UPDATE). Cochrane Database of Systematic Reviews 2022, Issue 3. Art. No.: CD003469. DOI: 10.1002/14651858.CD003469.pub4.~~

45. Thieme H, Morkisch N, Mehrholz J, Pohl M, Behrens J, Borgetto B, Dohle C. Mirror therapy for improving motor function after stroke. Cochrane Database of Systematic Reviews 2018, Issue 7. Art. No.: CD008449. DOI: 10.1002/14651858.CD008449.pub3. Accessed 04 August 2025.

46. Luvizutto GJ, Bazan R, Braga GP, Resende LADL, Bazan SGZ, El Dib R. Pharmacological interventions for unilateral spatial neglect after stroke. Cochrane Database of Systematic Reviews 2015, Issue 11. Art. No.: CD010882. DOI: 10.1002/14651858.CD010882.pub2. Accessed 04 August 2025.

47. das Nair R, Cogger H, Worthington E, Lincoln NB. Cognitive rehabilitation for memory deficits after stroke. Cochrane Database of Systematic Reviews 2016, Issue 9. Art. No.: CD002293. DOI: 10.1002/14651858.CD002293.pub3. Accessed 04 August 2025.

48. Gibson E, KohC-L, EamesS, BennettS, ScottAM, HoffmannTC.Occupational therapy for cognitive impairment in stroke patients. Cochrane Database of Systematic Reviews 2022, Issue 3. Art. No.: CD006430. DOI: 10.1002/14651858.CD006430.pub3.

49. Brady MC, Kelly H, Godwin J, Enderby P, Campbell P. Speech and language therapy for aphasia following stroke. Cochrane Database of Systematic Reviews 2016, Issue 6. Art. No.: CD000425. DOI: 10.1002/14651858.CD000425.pub4. Accessed 04 August 2025.

50. Bath PM, Lee HS, Everton LF. Swallowing therapy for dysphagia in acute and subacute stroke. Cochrane Database of Systematic Reviews 2018, Issue 10. Art. No.: CD000323. DOI: 10.1002/14651858.CD000323.pub3.

51. Linee Guida per la Diagnosi e la Riabilitazione dell’Eminegligenza Spaziale (neglect) nel paziente con ictus. Linea guida pubblicata nel Sistema Nazionale Linee Guida Roma, 15 gennaio 2024. https://www.iss.it/documents/20126/9185549/LG-C0012-SINP_Neglect.pdf/6a97b13c-0c4f-7769-15a2-067cd02c5c86?t=1705319915249

52. Linee Guida per la Valutazione e la Riabilitazione dell’Afasia nel paziente con ictus e nel paziente con Afasia Progressiva. Linea guida pubblicata nel Sistema Nazionale Linee Guida Roma, 19 dicembre 2023. https://www.iss.it/documents/20126/9185549/LG-C0011-SINP_Valutazione-riabilitazione-afasia-in+pz-con-ictus-e-afasia-prog.pdf/d8c0c53a-4aa7-485b-55f1-00ef784a6ddb?t=1702981161858

**S3_ Clinical assessments**

**Table 1. Upper limb impairment evidence-based clinical assessments**

| **Upper limb impairment** | |
| --- | --- |
| **Assessment** | **ICF-classification** |
| **Strength & Coordination** | |
| **ARAT**^95^ | **Body function** |
| **Fugl-Meyer UL**^19^ | **Body function** |
| **mRS**^42^ | **Activity**  **Participation** |
| **SIS**^43^ | **Body function**  **Activity**  **Participation** |
| **MRC**^96^ | **Body function** |
| **Spasticity** | |
| **MAS**^22^ | **Body function** |
| **~~Bradikynesia~~** | |
| **~~UPDRS~~**^~~97~~^ | **~~Body function~~**  **~~Activity~~** |
| **~~Tremor~~** | |
| **~~UPDRS~~** | **~~Body function~~**  **~~Activity~~** |
| **Sensation** | |
| **Fugl Meyer UL**^19^**: Sensory proprioception** | **Body function** |

**Table 2 Gait & balance impairment evidence-based clinical assessments**

| **Lower limb impairment** | |
| --- | --- |
| **Assessment** | **ICF-classification** |
| **Strength** | |
| **Fugl Meyer LL**^19^ | **Body function** |
| **mRS**^42^ | **Activity**  **Participation** |
| **SIS**^44^ | **Body function**  **Activity**  **Participation** |
| **FAC**^98^ | **Activity**  **Participation** |
| **Spasticity** | |
| **MAS**^22^ | **Body function** |
| **Gait & Balance** | |
| **Gait speed:6-min walk test, 10m walk test** | **Body function**  **Activity** |
| **~~UPDRS: Part 3~~**^~~97~~^ | **~~Body function~~**  **~~Activity~~** |
| **Berg balance scale**^41^ | **Body function** |
| **Timed Up and Go test** | **Body function**  **Activity** |
| **MotricityIndex/Trunk Control test** | **Body function** |

**Table 2.1 Instrumental assessment for motor impairment**

| **Motor Impairment** | |
| --- | --- |
| **Assessment** | **ICF-classification** |
|  | |
| **MEPs** | **Body function** |
| **Kinematic measures** | **Body function** |

**Table 3. Cognitive impairment evidence-based clinical assessments**

| **Cognitive impairment** | |
| --- | --- |
| **Assessment** | **ICF-classification** |
| **Screening Tools** | |
| **OCS**^54^ | **Body function** |
| **MOCA**^56^ | **Body function** |
| **MMSE** ^99^ | **Body function** |
| **~~UPDRS Part I~~**^~~100~~^ | **~~Body function~~**  **~~Activity~~** |
| **Spatial Neglect** | |
| **CBS** ^59^ | **Activity**  **Participation** |
| **BIT**^101^ | **Body function** |
| **Attention** | |
| **Trail Making Test (form A and B)**^97^ | **Body function** |
| **Attentional Matrices** | **Body function** |
| **Multiple Features Target Cancellation** | **Body function** |
| **Symbol Digit Modalities Test** | **Body function** |
| **SIS** ^44^ | **Activity**  **Participation** |
| **Memory** | |
| **Rey Auditory Verbal Learning Test** | **Body function** |
| **Prose Memory** | **Body function** |
| **Rey Complex Figure** | **Body function** |
| **Digit span** | **Body function** |
| **Spatial span (Corsi Test)** | **Body function** |
| **SIS** ^44^ | **Activity**  **Participation** |
| **Executive Functions** | |
| **Frontal Assessment Battery** | **Body function** |
| **Stroop Test** | **Body function** |
| **Weigl’s sorting test** | **Body function** |
| **Elithorn’s Perceptual Maze Test** | **Body function** |
| **Cognitive Estimation Task** | **Body function** |
| **Story-Based Empathy Task** | **Body function** |

**Table 4. Speech and language impairment evidence-based clinical assessments**

| **Speech and language impairment** | |
| --- | --- |
| **Assessment** | **ICF-classification** |
| **Screening Tools** | |
| **ELLM**^102^ | **Body function** |
| **SAND**^103^ | **Body function** |
| **Speech and Language** | |
| **AAT**^104^ | **Body function** |
| **ENPA**^104^ | **Body function** |
| **BADA**^105^ | **Body function** |
| **Protocollo di valutazione della disartria** | **Body function** |
| **Communication** | |
| **APACS**^106^ | **Body function**  **Activity**  **Participation** |
| **COAST**^107^ |  |
| **I-ASHA-FACS** |  |
| **SIS** |  |

**Table 5. Dysphagia evidence-based clinical assessments**

| **Dysphagia** | |
| --- | --- |
| **Assessment** | **ICF-classification** |
| **Functional** | |
| **DOSS**^108^ | **Body function**  **Activity** |
| **FOIS**^109^ | **Body function**  **Activity** |
| **SRS**^110^ | **Body function**  **Activity** |
| **Instrumental** | |
| **PAS**^111^ | **Body function** |

**S4_Glossary of Interventions**

**UPPER AND LOWER LIMB IMPAIRMENT INTERVENTIONS**

- **Action Observation**: A therapy where participants watch videos or demonstrations of movements to activate brain areas related to movement execution and programming and aid motor learning.
- **A FES (Functional Electrical Stimulation):** Use of electrical currents to activate muscles, often paired with EMG biofeedback to assist voluntary movement and motor re-education. It may also be used to treat muscle hypertonia, when electrical stimulation is applied to muscles that oppose the hypertonic ones, helping balance muscle activity and reduce abnormal tone. If the technology is not available, assistive intervention by the therapists, e.g. partial integration of force to execute a specific movement or task, may partially substitute FES.
- **Aerobic Training:** Exercises such as walking, cycling, or swimming that improve cardiovascular endurance and overall physical health.
- **Botulinum Toxin:** A neurotoxin injected into overactive muscles to temporarily block neuromuscular conduction, reducing muscle tone and improving range of movement.
- **Compensatory Strategies:** Techniques or adaptations that allow a person to perform tasks despite impairments, often by using the unaffected side, learning bimanual task for UL, using or assistive devices.
- **Enriched Environment:** A stimulating setting offering varied physical, social, and sensory activities to encourage cognitive and motor recovery leveraging increased brain plasticity.
- **Robot-Assisted Therapy:** robotic devices that train limb movement. They are divided into exoskeletons, i.e. wearable devices that integrate movement with various degrees of support, or end-effectors, i.e. robotic arms with an extremity (the end effector) that interacts directly with the end-user’s limb or body part. The degree of support ranges from full-support to assistive support, where the user voluntarily activates motion and the robot integrates strength to reach the range of motion. In settings with no robotic devices, conventional assistive/passive therapy substitutes the mechanical input.
- **~~Flexibility Training:~~** ~~Exercises designed to maintain or increase joint range of motion and reduce stiffness.~~
- **Inhibitory Postures:** Specific positions or postural strategies that help reduce muscle tone and inhibit muscle hypertonia.
- **Mirror Therapy:** A technique where a mirror reflects the unaffected limb, creating the illusion of movement in the impaired limb to stimulate brain recovery by activating the affected motor cortex.
- **mCIMT (Modified Constraint-Induced Movement Therapy):** A method that restricts use of the unaffected limb to encourage use of the impaired one, promoting neuroplasticity and functional improvement.
- **Motor Imagery:** Mental practice of movements without physical execution, engaging brain regions involved in motor control to support neuroplasticity and recovery.
- **Orthosis:** External devices such as braces or splints used to support, align, or biomechanically correct movement of limbs and joints.
- **Passive Mobilization:** Manual movement of a limb by a therapist or a robotic device to preserve range of motion and body image, and reduce stiffness. It is particularly useful when active movement is limited.
- **Progressive Resistance Training:** Strengthening exercises using gradually increasing resistance to build muscle strength and endurance.
- **Proprioceptive Training:** Activities designed to improve awareness of body position, movement, and balance through feedback from muscles and joints.
- **~~Relaxation Techniques:~~** ~~Methods such as deep breathing or guided imagery used to reduce stress, which may help manage tremors or intrusive motor symptoms.~~
- **Sensory Cueing (Visual, Auditory):** External prompts such as rhythmic sounds or lights used to guide timing and coordination of movement, particularly for gait retraining.
- **Sensory Stimulation:** Techniques that provide sensory input (touch, temperature, vibration, etc.) to enhance sensory processing and promote sensori-motor recovery.
- **Shaping Techniques:** Gradual motor skill-building through repetitive practice and reinforcement, often used in motor learning and behavioral therapies.
- **Stretching / Stretching Exercises:** Techniques to elongate muscles and tendons, commonly used to reduce tightness and hypertonia.
- **Task-Specific Practice:** Repetitive practice of meaningful, goal-directed tasks to enhance function through motor learning.
- **Treadmill Training:** Walking exercises on a treadmill, sometimes with body weight support, used to retrain gait.
- **Virtual Reality-Based Rehabilitation:** Interactive computer-based environments that simulate real-life tasks and provide feedback to enhance motivation and motor learning.
- **Visual & Vestibular Interventions:** Therapies targeting the visual and balance systems to improve orientation, coordination, and postural control.
- **Vibration Therapy:** Use of mechanical vibrations to stimulate muscles and nerves, potentially improving sensory input.

| **Intervention** | **Most‑cited Evidence (Cochrane or review)** | **Key Insight** |
| --- | --- | --- |
| **Action Observation** | Borges et al. (2022) Action Observation for Arm Rehabilitation after Stroke, Cochrane CD011887 – 158 citations | Small effects on arm function and larger on hand function; low‑certainty evidence. |
| **Functional Electrical Stimulation (FES)** | Eraifej et al. (2017) Systematic review of upper limb FES after stroke – 285 citations | Statistically significant modest benefits on ADL when applied early; overall evidence low quality. |
| **Aerobic Training** | Gezer H. (Cochrane Central Review CN‑01920115) Effect of Aerobic Exercise on Stroke Rehabilitation | Aerobic exercise may support neuroplasticity and function post-stroke, though high‑quality RCTs are limited. |
| **Botulinum Toxin (spasticity)** | Hoare et al. (2022) Cochrane CD003469 – upper limb in CP | Strong evidence in CP; for adult post‑stroke upper limb spasticity, systematic reviews show reduced tone but inconsistent functional gains. |
| **Mirror Therapy** | Thieme et al. (2018) Cochrane CD008449 – 888 citations | Moderate improvements in motor function, ADLs, and pain; maintained at 6 months. |
| **Enriched Environment / Sensory Stimulation / Visual & Vestibular / Sensory Cueing / Vibration; ~~Relaxation~~ / Inhibitory Postures; Orthosis; ~~Flexibility~~; Passive Mobilization; Shaping; Proprioceptive; Motor Imagery; Task‑Specific; Robot‑Assisted; Virtual Reality; Progressive Resistance** | No single Cochrane review. Included in broad Cochrane overview: Interventions to improve arm and hand function after stroke (Cochrane overview, 2014; 40 reviews) | Overall, interventions focusing on task-specific and repetitive functional training show benefit; robot‑assisted and VR approaches increasingly studied but high‑quality trials are limited |
| **mCIMT** | Meta-analsysis Hansen et al. (2025) | Modified constraint-induced movement therapy improves upper limb function in the acute and sub-acute stages of stroke recovery within inpatient hospital settings. SMD 0.94 based on low-quality evidence. Sustainability of improvements and the impact on activities of daily living remains uncertain. |
| **Treadmill training** | Cochrane Stroke review (2017, 44 RCTs); 2025 update (59 RCTs) | Improvements in walking speed, endurance, balance, and gait function post-stroke. Effect size is modest-to-moderate (SMD 0.5-0.7); greatest gains with high-duration, frequent training and body-weight support. |
| **Compensatory Strategies (assistive/prosthetics)** | Same Cochrane arm‑hand overview; Pollock et al. (2014) | Compensatory task-based approaches support functional improvement, particularly when personalized |

**COGNITIVE IMPAIRMENT INTERVENTIONS**

- **Hemi-field eye patching (binocular)**: A technique where one half of the visual field is occluded using a patch to encourage use and awareness of the neglected side.
- **Cueing**: External prompts (visual, auditory, or tactile) used to guide the patient’s attention toward the neglected space.
- **Arm activation therapy**: Repetitive, voluntary movement of the neglected limb to stimulate spatial awareness and sensorimotor integration.
- **Motion stimulation**: Use of dynamic stimuli (e.g. moving objects) in the neglected field to capture attention and promote orienting behavior.
- **Prism Adaptation Treatment**: A visuomotor intervention using prism goggles to shift visual input, helping recalibrate spatial perception toward the neglected side.
- **Visual scanning training**: Structured tasks that train patients to voluntarily shift their gaze and attention across the visual field, promoting exploration of neglected space.
- **Feedback and cueing (bottom-up approach)**: Use of sensory stimuli (e.g., flashing lights or sounds) to involuntarily draw attention to the neglected side.
- **Mental practice and imagery**: Patients imagine performing movements or scanning tasks, engaging attentional networks and promoting functional recovery.
- **Computer-based training**: Interactive programs that train cognitive functionsthrough cognitive tasks in a controlled digital format.
- **Paper & pencil training**: Traditional cognitive tasks using printed materials to improve cognitive functions.
- **Pharmacological (e.g., rivastigmine, amantadine, metilfenidate)**: Medications prescribed to enhance cognitive alertness and attention by modulating neurotransmitter activity.
- **Visual search task:** spatial exploration task to train sustained and selective attention.
- **Go-no-go task**: Two-condition task that require to inhibit a response in the no-go stage, used to train inhibitory control ability.
- **Tonic alertness training:** task designed to train alertness through repetitive tasks.
- **Trail making task**: Exercises involving structured cognitive tasks that target attention-switching to train divided attention.
- **Compensative training: use of external memories**: Techniques that teach patients to use tools like notebooks, alarms, and apps to compensate for memory deficits.
- **Psychoeducation (e.g., meta-memory)**: Structured education to improve awareness and understanding of one’s own memory strengths and limitations.
- **Mnemotechnic training (e.g., mental imagery, dual coding)**: Cognitive strategies that associate information with images or verbal-visual links to enhance recall.
- **Computer-based training (e.g., errorless learning, vanishing cues, spaced retrieval)**: Digital platforms delivering structured memory tasks with techniques that reduce errors and optimize recall through repetition and cue reduction.
- **Paper & pencil strategy training**: Memory enhancement using printed exercises that follow principles like errorless learning and spaced retrieval to train memory systematically.
- **Compensative training (e.g., self-instruction, mirror/video feedback, problem-solving procedure)**: Techniques that help patients compensate for deficits through guided instructions and structured routines.
- **Adaptive training (e.g., lists and diaries)**: Strategies that promote daily functioning through use of tools like to-do lists, planners, and schedules**.**
- **Restitutive training (e.g., goal management training, behavioral self-regulation, response inhibition)**: Interventions targeting underlying executive dysfunctions by restoring cognitive processes such as planning, monitoring, and impulse control.
- **Enriched Environment**: A stimulating setting offering varied cognitive, physical, and social activities to promote overall brain engagement and neuroplasticity.

| **Intervention** | **Most Cited Evidence (Cochrane or Review)** | **Key Insight** |
| --- | --- | --- |
| **Hemi‑field eye patching (binocular)** | Smania et al. (2013) review of eye patching RCTs (non‑Cochrane) | Mixed RCTs show some benefit for neglect; effects inconsistent and not Cochrane‑reviewed. |
| **Cueing (bottom‑up sensory cueing etc.)** | Longley et al. (2021) non‑drug treatments Cochrane CD003586 | Visual/auditory cues improve test neglect scores; unclear impact on functional ADLs. |
| **Arm activation therapy / Motion stimulation** | Longley et al. spatial neglect Cochrane CD003586 | Bottom‑up motor activities like limb activation show modest neglect reduction on tests. |
| **Prism Adaptation Treatment** | Longley et al. CD003586 Cochrane & meta‑analyses (e.g. Naito et al. 2025) | Modest short‑term improvement (SMD ≈ 0.5) in neglect tests; less evidence on ADL gains. |
| **Visual scanning training** | Longley et al. CD003586 Cochrane | Structured scanning training improves neglect tests; no proven ADL benefit. |
| **Mental practice / imagery** | Cognitive rehabilitation neglect review, Bowen et al., 2013 | Top‑down approaches (mental imagery, scanning) show limited evidence in RCTs. |
| **Computer training (go/no‑go, visual search)** | Longley et al. CD003586 & broader cancels tasks reviews | Mixed results; some improvement in digital neglect tasks; unclear generalization. |
| **Paper & pencil training (visual search, trail tasks)** | Longley et al. Cochrane CD003586 | Traditional scanning tasks improve test scores; no proven effect on daily functioning. |
| **Pharmacological (rivastigmine, amantadine, methylphenidate)** | Luvizutto et al.  van der Veen meta‑analysis on TBI | Very low‑quality evidence rivastigmine may slightly improve neglect; methylphenidate shows promise in TBI but not stroke neglect. |
| **Compensative training (external memory strategies)** | Memory rehabilitation Cochrane CD002293 (das Nair & Lincoln 2016) | Errorless/spaced retrieval and external aids yield immediate memory benefits; no long‑term ADL improvement. |
| **Psychoeducation (meta‑memory)** | Same memory Cochrane review CD002293 | Awareness-based strategies help subjective memory; limited durable functional effects. |
| **Mnemotechnic training (dual‑coding, imagery)** | Memory rehabilitation Cochrane CD002293 | Mnemonic strategies produce short-term gains in recall; limited spillover to daily life. |
| **Computer-based memory training (errorless learning, spaced retrieval)** | Memory review Cochrane CD002293 | Technique-specific training improves memory test scores; no evidence for sustained ADL gains. |
| **Paper & pencil memory strategy training** | Memory review Cochrane CD002293 | Similar outcomes to digital versions: short-term gains, no functional effect. |
| **Compensative executive strategies (self‑instruction, problem solving)** | Executive dysfunction review Cochrane CD008391 & recent meta‑analyses (post‑2015) | Cochrane found insufficient evidence; newer studies (e.g., goal management training) indicate small‑moderate benefits. |
| **Adaptive training (lists, planners)** | das Nair memory review CD002293 | External tools help immediate function; no long-term functional improvements shown. |
| **Restitutive training (GMT, behavioral self‑regulation)** | Executive dysfunction Cochrane CD008391 & meta‑analysis evidence | Cochrane inconclusive; more recent evidence supports small‑moderate lasting effects on ADLs. |
| **Enriched Environment** | Cochrane Review CD011879 (environmental enrichment) | Early evidence: improvements in well-being, activity levels; human trials are of limited quality—mostly preclinical or pilot. |

**SPEECH & LANGUAGE IMPAIRMENT INTERVENTIONS**

- **Speech Production Treatment:** Interventions focusing on improving articulation and fluency through repetitive practice of speech sounds, syllables, and words, often involving motor planning and feedback.
- **Phonomotor Treatment:** A therapy method that trains phoneme perception and production by combining motor and sensory tasks to enhance sound-level processing and generalization to word-level speech.
- **Elaboration Treatment:** Focuses on expanding verbal output by encouraging detailed responses and sentence construction, promoting richness and flexibility in language production.
- **Semantic Feature Analysis (SFA):** A naming therapy where patients describe features of a target word (e.g., category, function, appearance) to activate related semantic networks and facilitate word retrieval.
- **Word–Picture Verification:** A comprehension-based task where patients verify whether a spoken word matches a pictured object, targeting semantic understanding and word recognition.
- **Conversational Training (APL, PACE, Conversational Treatment):** A set of socially oriented therapies (e.g., PACE: Promoting Aphasics’ Communicative Effectiveness) that encourage natural conversation through multimodal communication and turn-taking strategies.
- **Neurolinguistics Training:** Therapies grounded in linguistic theory, focusing on grammar, syntax, and phonology to retrain specific linguistic components of speech and language processing.
- **Melodic intonation therapy (MIT):** A structured speech therapy technique that uses singing-like intonation and rhythmic tapping to help people with non-fluent aphasia produce words and phrases more easily, by activating undamaged areas of the brain, especially in the right hemisphere.
- **Compensatory Training (e.g., Multimodality Aphasia Therapy, M-MAT; PACE; CCA):** Emphasizes the use of alternative or augmentative communication modes (e.g., gestures, writing, drawing) to support verbal output, especially in cases of severe aphasia.
- **Compensatory (i.e., Augmentative Communication):** Involves using tools such as communication boards, speech-generating devices, or gestures to supplement or replace speech in individuals with severe speech intelligibility impairments.
- **Speech-Oriented Training:** Targets the improvement of speech production mechanisms (e.g., respiration, articulation, prosody) through exercises aimed at enhancing intelligibility and clarity of speech.
- **Communication-Oriented Training:** Focuses on optimizing communication effectiveness through environmental modifications, repair strategies, pacing techniques, and listener training to support functional interaction.

| Intervention | Most Cited Evidence (Cochrane or Review) | Key Findings |
| --- | --- | --- |
| **Speech Production Treatment** | Cochrane CD000425 *Speech and Language Therapy (SLT) for Aphasia*, Brady et al. (2016) – ~1,400 citations | SLT overall benefits expressive language and speech production; no evidence favoring one speech-specific method over another. |
| **Phonomotor Treatment** | Included as part of SLT comparison in Cochrane CD000425 | SLT types not differentiated; specific evidence for phonomotor approaches are not isolated in Cochrane. |
| **Elaboration Treatment** | General discourse-focused SLT (Dipper et al., 2021) systematic review | Discourse treatments (e.g. elaboration) show gains in word- and clause-level output; limited high-quality trials and small samples. |
| **Semantic Feature Analysis (SFA)** | Efstratiadou et al. (2018) SR, and Maddy et al. (2014) Ann. Phys. Rehabil. Med. | SFA reliably improves naming of trained items, with small to large effect sizes; generalization to untrained items limited. |
| **Word–Picture Verification** | Part of SLT RCTs within Cochrane CD000425 | Treated within broad SLT; no discrete evidence isolating verification tasks. |
| **Conversational Training (PACE, APL, Conversational Treatment)** | Simmons‑Mackie et al. (2014) review; supported by SLT evidence in Cochrane CD000425 | Conversational methods show potential for functional communication; enclosed within SLT evidence but few high-quality RCTs specific to PACE. |
| **Neurolinguistics Training (grammar/syntax/phonology)** | No standalone Cochrane; considered as part of SLT in CD000425 | SLT theories not distinguished; no RCT-level differentiation for neurolinguistic-specific approaches. |
| **Neurolinguistics: Melodic Intonation Therapy (MIT** | Multi-level meta-analysis from Popescu et al. (2022) | MIT produces a small-to-moderate effect on validated non‑communicative expressive language outcomes (repetition tasks); effect sizes reported in non‑RCT studies were roughly five to six times larger (≈ 2.0), likely due to methodological bias and spontaneous recovery |
| **Compensatory Training (e.g. M‑MAT, PACE, CCA)** | AAC/systematic review of compensatory SLT and tools (Huang et al., 2021); part of SLT in Cochrane CD000425 | AAC and compensatory modalities support communication participation; formal device studies show mixed results; often included under broader SLT umbrella. |
| **Compensatory/Augmentative Communication** | Cochrane CD000425 acknowledges augmentative/comprehensive SLT interventions | Augmentative strategies improve interaction for severe aphasia; limited RCT data, some pilot evidence supports memory aid use. |
| **Speech‑Oriented Training** | Encompassed in Cochrane CD000425 SLT interventions | Speech-focused training (e.g., articulation drills) effective as part of SLT but not separately studied in Cochrane. |
| **Communication‑Oriented Training** | Included in Cochrane CD000425 (environmental/partner training) | Communication partner training improves interaction outcomes; environmental strategies suggested but limited RCT comparison. |

**DYSPHAGIA INTERVENTIONS**

- **Compensatory Strategies**: Involves immediate safety-focused techniques such as postural maneuvers (e.g., chin tuck, head turn), diet texture modification, and patient/family education to reduce aspiration risk and improve swallowing efficiency without changing the underlying physiology.
- **Behavioral Treatment (Swallowing and Rehabilitative Exercises)**: Aims to improve the strength, coordination, and timing of swallowing muscles through exercises such as effortful swallow, Mendelsohn maneuver, Shaker exercise, or tongue resistance tasks, often tailored to the patient's specific deficits.
- **Neurostimulation**: Includes techniques such as transcranial magnetic stimulation (TMS), transcranial direct current stimulation (tDCS), neuromuscular electrical stimulation (NMES), and pharyngeal electrical stimulation, applied to enhance neural plasticity and promote functional recovery of swallowing—often used with or without biofeedback.
- **Tracheostomy Tube Weaning**: A gradual process of reducing or removing the tracheostomy tube in medically stable patients, coordinated with respiratory and swallowing teams, to restore upper airway function and improve swallowing safety and efficiency.

| **Intervention** | Most Cited Evidence (Cochrane or Review) | **Key Findings** |
| --- | --- | --- |
| **Compensatory Strategies** (postural maneuvers, diet texture, education) | Cochrane Bath et al. (2018) CD000323 | Moderate-quality evidence that combined compensatory strategies, diet modification, and behavioral interventions may reduce dysphagia incidence (OR ~0.42), shorten hospital stay (−2.9 days), and lower chest infection/pneumonia, though outcomes on aspiration severity and mortality are inconsistent. |
| **Behavioral Treatment (swallowing exercises: effortful swallow, Mendelsohn, Shaker, tongue resistance)** | Same Cochrane review Bath et al. (2018) CD000323 | Very low- to moderate-quality evidence that behavioral swallowing interventions can improve swallowing ability (SMD ≈ –0.66), reduce dysphagia and infections; evidence is heterogeneous and intervention-specific effects unclear. Recent real-world observational study (2024) also supports benefit in nasogastric-fed acute stroke patients |
| **Neurostimulation (TMS, tDCS, NMES, PES)** | Cochrane review (Bath et al. 2018) includes these but with low‑quality evidence. Plus network meta-analyses: Chiang et al. (2019) He et al. (2022) tDCS meta‑analysis | Neurostimulation shows promise: NMES and tDCS may improve swallowing ability and quality of life; rTMS shows very large SMD (~5) for swallowing function in a single network review. However, overall evidence across meta-analyses remains moderate-to-low quality and highly variable in protocols. |
| **Tracheostomy Tube Weaning** | Narrative/systematic reviews and expert consensus (e.g. Skoretz et al. 2020; Gallice et al. 2023 algorithmic approach | No Cochrane review exists; evidence from observational and consensus literature suggests multidisciplinary, protocol-driven weaning may aid dysphagia recovery and decannulation, but well-controlled RCT data are lacking. |
